# A feasibility study of microwave therapy for precancerous actinic keratosis†

**DOI:** 10.1111/bjd.18935

**Published:** 2020-03-23

**Authors:** D.N. Jackson, F.J. Hogarth, D. Sutherland, E.M. Holmes, P.T. Donnan, C.M. Proby

**Affiliations:** ^1^ Department of Dermatology NHS Tayside Ninewells Hospital Dundee DD1 9SY Scotland UK; ^2^ Tayside Clinical Trials Unit University of Dundee DD1 9SY Scotland UK; ^3^ Clinical Research Centre NHS Tayside Ninewells Hospital Dundee DD1 9SY Scotland UK; ^4^ Dundee Epidemiology and Biostatistics Unit, Population Health and Genomics School of Medicine University of Dundee DD1 9SY Scotland UK; ^5^ Molecular and Clinical Medicine Ninewells Hospital & Medical School University of Dundee DD1 9SY Scotland UK

## Abstract

**Background:**

Actinic keratosis (AK) is a common premalignant skin lesion that can progress to cutaneous squamous cell carcinoma (cSCC). Microwave therapy is an established cancer treatment and has been used for plantar viral warts.

**Objectives:**

To evaluate the efficacy and feasibility of microwave as a treatment for AK.

**Methods:**

Stage I was a dose‐setting study, in which seven participants had the dielectric properties of 12 thick and 22 thin AKs assessed for optimization of the microwave dose used for treatment in Stage II. Stage II was a randomized, internally controlled trial evaluating 179 AKs in 11 patients (93 treated, 86 untreated controls) on the scalp/forehead or dorsal hand. Participants received one treatment initially and a repeat treatment to unresolved AKs at week 4. The response was assessed at six visits over 4 months. The primary outcome was partial or complete resolution of the treated AKs.

**Results:**

A significantly higher proportion of treated AK areas responded than untreated (90% vs. 15%; *P* < 0·001). Thin AKs were more responsive than thick AKs. The site did not affect efficacy. Pain was severe, but brief (80% reported pain lasting ‘a few seconds only’). Adverse effects were minimal (erythema, *n* = 6; flaking, *n* = 3; itch, *n* = 3). All participants who would chose microwave therapy over their current treatment cited the shorter discomfort period.

**Conclusions:**

Microwave therapy is a portable, safe and effective treatment for AK. An easy‐to‐deliver, acceptable therapy for AK is attractive as a prevention strategy. While these results are promising, a larger randomized controlled trial is needed against an effective comparator to confirm clinical efficacy and patient acceptability.

**What is already known about this topic?**

- Actinic keratoses (AKs) are common precancerous skin lesions.
- Successful treatment of AK can prevent cutaneous squamous cell carcinoma (cSCC).
- Most topical therapies for AK require repeated application over weeks and drive local skin inflammation, leading to poor compliance.
- An easy‐to‐deliver and effective treatment for AK, suitable for use in primary care, could reduce cSCC.

**What does this study add?**

- Microwave therapy is a feasible, effective treatment for AK.
- Ninety per cent of treated AKs showed full or partial resolution at 120 days post‐treatment.
- Microwave therapy was painful, but the pain was short‐lived (seconds) and this short discomfort period was cited as the main reason that microwave was preferred to their current treatment.

**Linked Comment:** Samimi and Kelleners-Smeets. Br J Dermatol 2020; 183:197–199.

## Abstract 2

**Linked Comment:** Samimi and Kelleners-Smeets. Br J Dermatol 2020; 183:197–199.

Plain language summary available online

Actinic keratosis (AK) is a common precancerous skin lesion found on light‐exposed sites in older fair‐skinned individuals with prevalence rates of 23·5% in the Dutch population over 50 years of age.^1^ AKs are precursors to cutaneous squamous cell carcinoma (cSCC), which has doubled in incidence in a decade due to ageing populations and increased ultraviolet radiation exposure.^2^ The individual risk of progression to cSCC is low,^3^ but 65% of cSCCs on the head and neck arise from AK.^4^ A double‐blind, randomized clinical trial (RCT) of 5% fluorouracil cream (5‐FU) showed a 75% risk reduction for development of cSCC in the year following treatment [95% confidence interval (CI) 35–91%; *P* < 0·002].^5^ This pivotal study suggests that annual treatment of AK should reduce the incidence of cSCC. Multiple field treatments for AK exist, such as 5‐FU, imiquimod 5% cream, diclofenac 3% gel, photodynamic therapy (PDT) and lesion‐directed therapy like liquid nitrogen (cryotherapy).^6^ Many AK treatments require dedicated application over weeks and drive significant inflammation. Furthermore, many AK sufferers are elderly and would find compliance easier with a lesion‐directed treatment.

Microwave therapies are established within oncology for ablative treatment of internal malignancies.^7^, ^8^ Microwave energy has shown promise in the treatment of recalcitrant plantar viral warts.^9^ This study used a CE‐marked microwave medical device (Swift^®^ Microwave Tissue Ablation System, Emblation Ltd, Alloa, UK). The applicator of the Swift^®^ device delivers microwave energy to the skin at a diameter up to 6 mm and depth of 2–6 mm depending on dosage. The electromagnetic waves excite water molecules, driving localized hyperthermia^10^ and accelerated chemical kinetics.^11^ Depending on dose, the treatment can have an ablative destructive or subablative nondestructive effect.

Here we report a first‐in‐human, two‐stage feasibility study of microwave therapy for the treatment of AK on the bald scalp, forehead or dorsal hand.

For Stage I, the objective was to determine the dielectric properties of AK for optimization of the Swift^®^ device microwave parameters to deliver a subablative dose of energy.

Stage II was a single‐site, randomized, internally controlled trial to evaluate the efficacy, long‐term resolution, safety and feasibility of microwave as a treatment.

## Materials and methods

### Study design and participants

This randomized, internally controlled, feasibility study of microwave therapy for the treatment of AK (NCT03483935) was conducted at Ninewells Hospital & Medical School, Dundee, UK, from January 2018 until April 2019. The study was co‐sponsored by the University of Dundee and NHS Tayside (approved December 2017) and was reviewed and approved by the East of Scotland Research Ethics Service (18/ES/0008, January 2018). Patients with AKs on the forehead, bald scalp or dorsal hands were recruited from the dermatology department, NHS Tayside. All participants provided written informed consent.

Inclusion criteria were age over 18 years with a minimum of six AKs on both the right and left side of the forehead/scalp or dorsal hand, able to give informed consent and perform study assessments. Exclusion criteria were AKs sited on the lip or ear, confluent AKs with field change, implantable cardioverter‐defibrillator, pacemaker or other implantable device, metal implants at the site of microwave treatment, known intolerance to microwave, unstable comorbidities (including cardiovascular disease, active malignancy, inflammatory arthritis) or participation in another interventional study.

Sample‐size calculations were based on 100 AKs (50 treated; 50 untreated, mapped and followed), with on average 10 per participant. In a paired analysis with McNemar's test, the power is 80% to detect a difference in proportion > 25% complete or partial resolution of 0·33. Repeated measures of AKs over six visits will give 300 paired measurements. Even with a smaller mean number of visits (i.e. three), the number of pairs is increased by the inflation factor to 55 assuming intraclass correlation coefficient of 0·05. Hence, with a mean of three visits (i.e. 150 paired measurements), power would be 80% to detect a difference of 0·2 between treated and untreated in proportion > 25% complete or partial resolution.

### Microwave dose

For Stage I, seven participants had the relative permittivity, conductivity and loss tangent of their AK measured. They did not receive any microwave dose. A median of five measurements per patient were taken, of which 22 (65%) were for thin AKs (Olsen grade 1 or 2) and 12 (35%) were for thick AKs (Olsen grade 3).^12^ The data allowed calculation of the energy required to raise the tissue temperature into the subablative region.^13^ Subablative doses are generally considered to be below 50 °C.^13^ A microwave treatment dose of 5 watts (5W) delivered for 3 s and repeated three times with 20‐s gaps was chosen.

When the 5W dose was delivered to the first two participants, it caused severe pain and some ulceration/scabbing, suggesting it was ablative rather than subablative. Modelling performed by Emblation, the manufacturer of Swift^®^, using data from the permittivity study and a finite element solution of the bioheat transfer equation suggested that 4W for hyperkeratotic ‘thick’ AK (Olsen grade 3) and 3W for nonhyperkeratotic ‘thin’ AK (Olsen grades 1 and 2) would still provide a therapeutic subablative tissue temperature, with the benefit of being more tolerable (Table S1; see Supporting Information).^14^ Consequently, the protocol was amended to reflect this reduction in dose. The protocol change was submitted as a substantial amendment and approved by the East of Scotland Research Ethics Service prior to continuing the study.

### Study assessments

At the screening visit, participants consenting to Stage II had the treatment site (scalp/forehead or hands) chosen by the Chief Investigator based on a clinical decision. AKs were mapped using an acetate grid and photographed using an agreed protocol to aid assessments at later visits. Participants underwent a general examination to exclude significant comorbidities and coincidental skin cancer. Participants were randomized to treatment to the left or right side. The randomization system used was TRuST, a good clinical practice‐compliant Tayside Clinical Trials Unit Interactive Web Response System. No stratification or minimization was used. One AK on the treatment side was preselected at screening for biopsy at visit 4. The treatment side received microwave therapy, with mapped AKs on the contralateral side observed as untreated controls. The probe was placed in the centre of the AK for each treatment and AKs larger than the treatment probe were not excluded as it was unclear whether benefit might extend to adjacent areas of AKs through local inflammatory or immunological effects. We wished to test whether AKs larger than the applicator tip would resolve completely or only partially. Participants were asked to rate their pain immediately following treatment and 30 min later.

Participants attended for six follow‐up visits at 1, 2, 4, 6, 8 and 16 weeks post‐microwave treatment. At each visit AKs were assessed by the Chief Investigator or delegate and scored as completely resolved, partially resolved or unchanged. Participants were asked about local adverse events (itching, stinging, soreness, redness, flaking, ulceration, pus) and whether these were mild or severe. Photographs were taken of treated and control AKs at each visit to aid assessment. There were three telephone follow‐ups on weeks 3, 5 and 7 to assess adverse events following treatment and biopsy.

A pre‐assigned, treated AK was biopsied (4‐mm punch biopsy) at 2 weeks post‐treatment (visit 4) for histology and transcriptome studies.

At week 4, there was the option to repeat treatment to any previously treated but unresolved AK.

The final visit and AK assessment took place at 4 months (day 120). Participants were asked to complete a self‐assessed health index about their experience of microwave therapy.

### Statistical analysis

The primary outcome, resolution of the treated area of the AK lesion, was predetermined as either partial (resolution of the area covered by the microwave probe, but with a rim of persistent AK) or full resolution (complete resolution of the entire AK) over all time periods. Response was assessed at visits 3 (day 8), 4 (day 15), 6 (day 28), 8 (day 42), 10 (day 60) and 11 (day 120). Mixed‐effects logistic regression models analysed the effect of microwave therapy with random effects for participant and visit (≤ 6 per participant). Each visit was analysed as a categorical variable as they were spaced unequally in time. Variables representing sex, age, skin site (hand/scalp) and AK subtype (thick/thin) are included as covariates.

The secondary outcome of long‐term response was assessed using data from visits 10 and 11 (days 60 and 120) only. Again, nonlinear models were utilized with random effects for participant and visit (≤ 2 per participant) and covariates for sex, age, skin site (hand/scalp) and AK subtype (thick/thin).

Pain during and following treatment were secondary outcomes. SAS Enterprise Guide software (version 6·1, SAS Institute Inc., Cary, NC, USA) was used for all statistical analyses.

## Results

### Study participants

**Figure 1:**
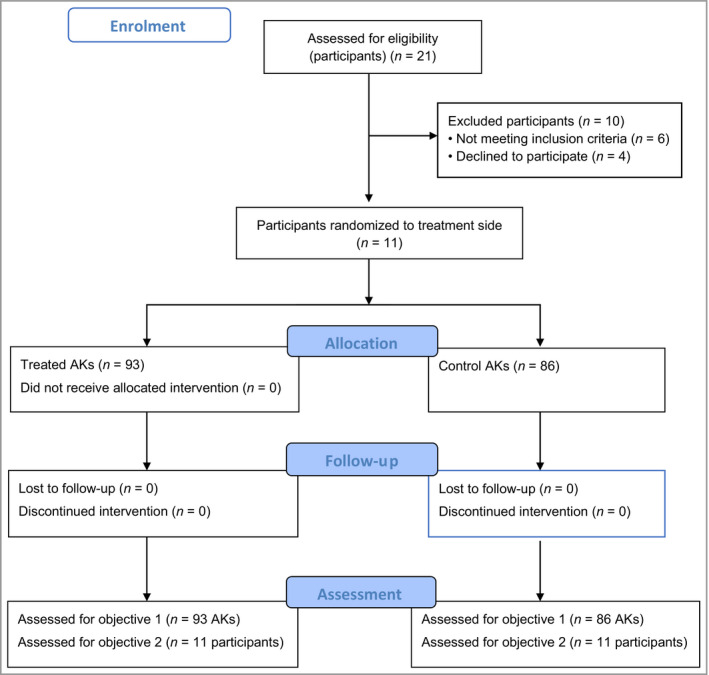
CONSORT diagram.

Eleven participants, seven male and four female, gave informed consented and were randomized to Stage II. The Consort flow diagram is illustrated in Figure 1. Participant demographics and cancer history are shown in Table 1. Ten of 11 (91%) participants had received prior treatments with both cryotherapy and 5‐FU cream. Additional treatments in order of frequency were 3% diclofenac gel (Solaraze) (64%), 5% imiquimod cream (Aldara) (55%), ingenol mebutate gel (Picato) (45%), surgery (45%), PDT (36%) and 5‐FU 0·5% and salicylic acid 10% topical solution (Actikerall) (27%) as detailed in Table S2 (see Supporting Information).

**Table 1:** Baseline characteristics for 11 participants

Variable	Statistic/status	Summary
Age (years)	*n*	11
	Mean (SD)	78 (6)
	Median	78
	Range	62–88
Sex	Female	4 (36%)
	Male	7 (64%)
History of skin cancer	Yes	10 (91%)
	No	1 (9%)
History of other cancer	Yes	0 (0%)
	No	11 (100%)

### Microwave dose

Following permittivity studies (Stage I), the microwave dose was chosen and delivered as described in Materials and methods. The first two participants in Stage II received a 5W dose for 3 s repeated three times to each treated AK, but the subsequent nine participants received 3W 3‐s doses to thin AKs (Olsen grades 1 & 2) and 4W 3‐s doses to thick AKs (Olsen grade 3).

### Treatments and biopsies

Eleven participants were randomized to the RCT and 179 AKs (93 treated, 86 untreated) assessed (Figure 1). All participants completed treatment as planned and at follow‐up visit 10 (day 60) one participant's treated AKs could not be assessed due to an unrelated hospital admission (the hand was bandaged for an *in situ* cannula). Ten participants underwent a second treatment on day 28 (visit 6), with 51 of 93 (55%) treated AKs receiving a second treatment. One of the participants who had received the 5W dose declined repeat treatment due to pain. All biopsies of pre‐assigned AKs were undertaken at day 15, 2 weeks after the first treatment.

Biopsies of AKs treated with 5W (*n* = 2) showed dermal fibrosis, mixed acute and chronic inflammatory infiltrate, and some reactive squamous metaplasia of eccrine ducts. Any epidermal dysplasia was mild. Six of the remaining nine AK biopsies showed some inflammation with mild‐to‐moderate epidermal dysplasia in the majority, although three of nine noted moderate‐to‐severe epidermal dysplasia consistent with persistent AK.

### Effectiveness of treatment

**Figure 2:**
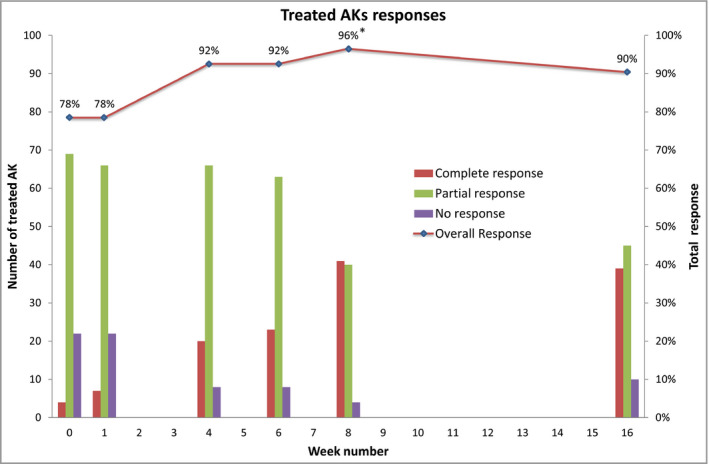
Percentage of treated actinic keratoses (AKs) that responded by week.

**Figure 3:**
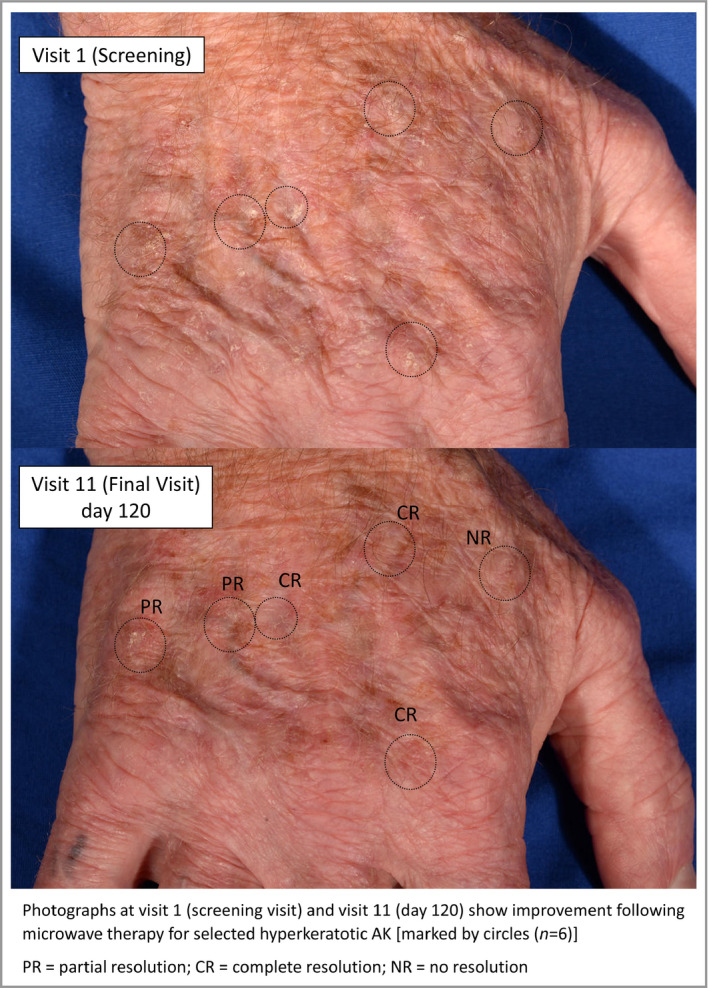
Photographs at visit 1 (screening visit) and visit 11 (day 120) show improvement following microwave therapy for selected hyperkeratotic actinic keratoses [marked by circles (*n* = 6)]. CR, complete resolution; NR, no resolution PR, partial resolution.

Overall response rates (including both partial and complete resolution) for treated AKs were 78% at visit 3 (day 8), rising to 90% at visit 11 (day 120) (Table 2, Figure 2), compared with 2% at visit 3 and 15% at visit 11 for untreated AK. The results of a nonlinear repeated measures model of resolution found a significantly higher proportion of AKs treated with microwave therapy to have fully or partially resolved compared with untreated control AKs (odds ratio 154, 95% CI 75–317, *P* < 0·001, Table 3). The magnitude of the odds ratio reflects the sustained resolution of treated AKs across all visits (Table 2, Figure 2). The photographs in Figure 3 show examples of partial and complete resolution.

**Table 2:** Summary for primary endpoint by visit for AK lesions

Visit	Treated			Not treated		
	Lesions, *n*	*n* (%)	Resolution	Lesions, *n*	*n* (%)	Resolution
Visit 3 (day 8)	93	73 (78)	4 CR, 69 PR	86	2 (2)	1 CR, 1 PR
Visit 4 (day 15)	93	73 (78)	7 CR, 66 PR	86	7 (8)	1 CR, 6 PR
Visit 6 (day 28)	93	86 (92)	20 CR, 66 PR	86	9 (10)	2 CR, 7 PR
Visit 8 (day 42)	93	86 (92)	23 CR, 63 PR	86	9 (10)	3 CR, 6 PR
Visit 10 (day 60)^a^	84	81 (96)	41 CR, 40 PR	86	11 (13)	5 CR, 6 PR
Visit 11 (day 120)	93	84 (90)	39 CR, 45 PR	86	13 (15)	6 CR, 7 PR

a The clinical investigator was unable to assess nine treated AKs on one participant at visit 10 due to the participants’ hand being cannulated for other medical treatment during an unrelated inpatient admission.

CR, complete resolution; PR, partial resolution

**Table 3:** Odds ratios (OR) from the mixed model for actinic keratosis response

Variable	OR	95% CI	*P*‐value
Treatment vs. placebo	154	75–317	< 0·001
Visit			< 0·001
Visit (3 vs. 11)	0·25	0·1–0·58	
Visit (4 vs. 11)	0·33	0·14–0·75	
Visit (6 vs. 11)	0·87	0·42–1·78	
Visit (8 vs. 11)	0·87	0·42–1·79	
Visit (10 vs. 11)	1·25	0·65–2·42	
Age (+ 1 year)	1·03	0·97–1·08	0·33
Sex: female (ref, male)	0·56	0·26–1·21	0·14
Scalp site (ref, hand)	1·68	0·78–3·61	0·18
Thin subtype (ref, thick)	3·47	1·8–6·66	< 0·001

CI, confidence interval

**Figure 4:**
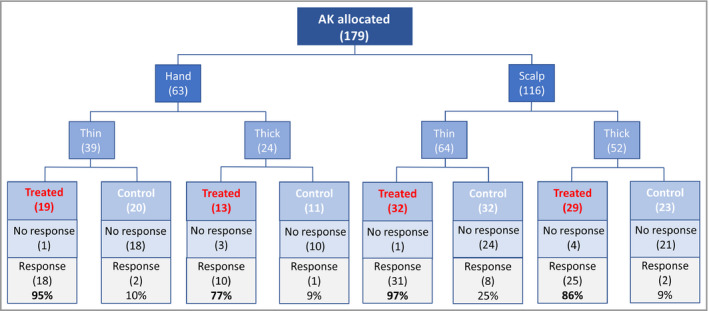
Summary tree.

The type of AK (thick vs. thin) was associated with response (Table 3, Figure 4). Thin AKs had a higher response rate than thick AKs, but similarly, in the untreated group, thin AKs were more likely to resolve spontaneously (Figure 4).

### Participant‐reported pain and safety

Most participants reported ‘moderate’ or ‘severe’ pain during treatment and all participants reported no pain after 30 min (Table 4). Eighty per cent of participants reported pain lasting a few seconds only; 20% reported pain lasting up to 5 min. Redness (*n* = 6), flaking (*n* = 3) and itching (*n* = 3) were reported as adverse events. There were no unexpected or serious side‐effects. Most participants preferred microwave or had no preference when comparing their experience of microwave with previous treatments (Table 5).

**Table 4:** Summary of pain during treatment

Variable	Visit 2 participants, *n* (%)	Visit 6 participants, *n* (%)
Pain during treatment		
Mild	7 (8)	5 (10)
Moderate	32 (34)	27 (53)
Severe	54 (58)	19 (37)
Duration of pain		
Few seconds	7 (64)	8 (80)
Up to 5 min	2 (18)	2 (20)
Up to 10 min	1 (9)	0 (0)
Up to 20 min	1 (9)	0 (0)
Still sore at 30 min	0 (0)	0 (0)

**Table 5:** Patient experience

	Week 10	Week 11
*Choice of another AK treatment*		
Prior treatment	1	2
Microwave	4	6
No preference	6	3
*Reason for choice* a		
Less pain	1	1
Shorter discomfort period	3	6
Fewer side‐effects	3	4
Other	2	1
*Pain if second treatment*		
More painful	1	0
Less painful	5	5
No difference	4	5
Not applicable	1	1
*Worry about treatment*		
Yes	0	1
No	11	10

AK, actinic keratosis

a Not all participants gave a reason for their choice and some participants chose more than one reason for their choice

## Discussion

This first‐in‐human study suggests that microwave therapy might be a promising treatment for AK, with 90% of AKs showing resolution of the treated area at 120 days post treatment (Table 2). This was highly significant (*P* < 0·001) and was more effective for thin than for thick AKs (*P* < 0·001), as has been noted with most other therapies, including PDT.^15^, ^16^ Our study included hyperkeratotic AKs and acral sites, both of which are associated with higher rates of treatment failure.^6^, ^17^

AKs larger than the microwave probe diameter were not excluded in this study. Many larger AKs demonstrated complete resolution of the central area under the applicator tip, but with a rim of persistent AK outside this treatment area and these lesions were recorded as a partial response despite resolution of the treated area. Therefore, rates of complete resolution in this study appear to be relatively modest, with 42% of AKs showing complete resolution at 120‐day follow‐up. In future studies, adopting a stepwise overlapping delivery of treatment across the whole surface of the AK might lead to higher rates of complete resolution. This approach has been used successfully for plantar warts.^9^ Seven per cent of untreated AKs had spontaneously resolved by visit 11, which is similar to that reported in other studies^18^ and demonstrates the importance of an internal control.

The participants in this study were representative of the patient population with AK, with a median age of 78 years and majority male (64%). An increased prevalence of AK with advancing age and male sex has been noted in both primary^19^ and secondary care.^20^ Importantly, there was no difference in response with sex, age or skin site. In contrast, many alternative therapies have demonstrated reduced efficacy on acral sites.^6^, ^17^

One week after initial treatment (visit 3), 78% of AKs showed a response, which rose to 92% by 4 weeks (visit 6), which may suggest that induction of an immune response promotes clearance. A similar effect was seen with treatment to plantar warts.^9^ This implies that there may be a possibility of ‘field’ benefit with microwave therapy and in this study, as demonstrated in Figure 3, the post‐treatment appearance did show a general improvement in addition to specific resolution (partial or complete) of individual AKs. Nonetheless, microwave therapy should be considered a lesion‐directed therapy rather than a field‐directed therapy and any subsequent examination in a head‐to‐head comparison with current AK therapies should include a cryotherapy arm as well as a topical 5‐FU or imiquimod arm.

The main side‐effect was pain. The first two participants, treated with 5W doses, found the treatment very painful and one participant declined repeat treatment at 4 weeks. This higher dose appears more efficacious with 100% of AKs showing a response to the 5W dose compared with 88% with the 3W or 4W dose (Table S3; see Supporting Information). All participants were included in the overall statistical assessment. The subsequent lower doses (3W or 4W), delivered after an amendment to the protocol, were well tolerated. Participants described pain as ‘minimal’ initially, but ‘very painful’ for the final 1 s of treatment. While 58% of participants described pain as severe at the first treatment, this reduced to 37% with the second treatment, and most (80%) reported the pain as lasting a few seconds only (Table 4). This reduction may be due to greater expectation with repeat treatment. All participants eligible for the study completed it and none of the participants who received the lower microwave dose with the revised protocol declined a second treatment, suggesting that this was tolerable, despite treatment of up to 10 AKs per treatment. Rarely did pain last longer than 5 min (Table 4) and severe pain never lasted more than a few seconds. This short duration undoubtedly makes the treatment more tolerable. When surveyed about their patient experience on day 120, six patients would choose microwave treatment over their current AK therapy, and all cited a shorter discomfort period as a reason (Table 5). Pain is common with existing treatments such as cryotherapy and PDT; however, patients often accept this if a treatment is effective.^21^

Other adverse events following microwave treatment were minimal [erythema (*n* = 6), flaking (*n* = 3) and itch (*n* = 3)]. This contrasts favourably with treatments like 5‐FU or 5% imiquimod cream, which cause significant inflammation including swelling, erosions, crusting and blistering.^22^ Diclofenac 3% in hyaluronic acid gel causes less severe local skin reactions^23^ and is a treatment favoured by general practice in the UK.^24^, ^25^ However, its long treatment duration (3 months) may reduce compliance.

There were study limitations. This was a small study with 11 participants, but analysis was per AK and 179 AKs were assessed, increasing the power. Participants were recruited from secondary care so had relatively severe AK. Sufficient ‘thick’ and ‘thin’ AKs were treated to analyse these subgroups independently. While the side to receive treatment was randomized, the assessors were not blinded. Blinding was not feasible as only two clinicians were involved in both treatment delivery and follow‐up. Furthermore, erythema from the treatment and, at later visits, the biopsy scar, would reveal the treatment side. As a first‐in‐human study, the effects of treatment on AKs were not known. As such, both partial and complete resolution of individual AKs were assessed and we have reported the response rates by complete, partial or none in Table 2 and Figure 2. Due to the lack of overlapping or stepwise treatment over the entire surface of larger AKs, this study may underestimate the potential for complete resolution. There was minimal missing data.

The microwave device used is portable and safe and does not require the impractical storage infrastructure of cryotherapy. Minimal training is needed and multiple lesions can be treated in a single session. These factors make it particularly suitable for primary care where AKs are prevalent. Of 2844 consecutive patients enrolling with a general practitioner in Switzerland, 23% had AK.^19^ Given the mounting evidence that treatment of AK can prevent cSCC,^5^ a treatment that can be delivered in primary care, possibly when the patient is attending for another reason, may be effective at reducing the burden of cSCC. While these results are promising, a larger RCT is needed against an effective lesion‐directed comparator such as cryotherapy, as well as a field treatment, to confirm clinical efficacy and patient acceptability.

## Supporting information


**Table S1** Results of Stage I modelling.
**Table S2** Previous therapies and preference by dose.
**Table S3** Summary for primary endpoint by treatment protocol.

**Powerpoint S1** Journal Club Slide Set.

**Video S1** Author video.
