# Supplementary material for: A feasibility study of microwave therapy for precancerous actinic keratosis
Source: Br J Dermatol. 2020 Mar 23;183(2):222–30. doi: 10.1111/bjd.18935 (PMC7496712; doi:10.1111/bjd.18935)
Supplement: Supplementary file 1 — Table S1 Results of Stage I modelling. Table S2 Previous therapies and preference by dose. Table S3 Summary for primary endpoint by treatment protocol. [file BJD-183-222-s001.docx]

**Supplementary Table S1: Results of Stage** I **modelling**

| AK Type | AK Location | Tissue Temperature* of lesion as calculated by modelling | | |
| --- | --- | --- | --- | --- |
|  |  | 5W 3s | 4W 3s | 3W 3s |
| Thick | Hand | 46.9°C | **44.3**°C | 41.8°C |
| Thick | Scalp | 46.9°C | **44.3**°C | 41.8°C |
| Thin | Hand | 47.8°C | 45.1°C | **42.4**°C |
| Thin | Scalp | 50.7°C | 47.7°C | **44.3**°C |

* Italics = initial treatment protocol (participants 1-2); **Bold** = amended treatment protocol (participants 3-11)

**Supplementary Table S2: Previous therapies and preference by dose**

|  | previous therapy | | | | | | | | |  | Future preference^a^ | |  |
| --- | --- | --- | --- | --- | --- | --- | --- | --- | --- | --- | --- | --- | --- |
| Participant | cryo | Surgery | Solaraze^b^ | Efudix^c^ | Aldara^d^ | Actikerall^e^ | Picato^f^ | PDT | Other | Dose (w) | v10 choice | v11 Choice | No of treatments |
| 1 | no | no | yes | yes | no | yes | no | no | no | 5 | none | none | 2 |
| 2 | yes | yes | yes | yes | yes | no | no | no | no | 5 | current | current | 1 |
| 3 | yes | no | yes | no | no | no | yes | no | no | 3-4 | none | none | 2 |
| 4 | yes | no | yes | yes | yes | no | no | no | no | 3-4 | none | none | 2 |
| 5 | yes | no | no | yes | no | no | yes | no | no | 3-4 | MW | current | 2 |
| 6 | yes | yes | yes | yes | yes | yes | yes | no | yes | 3-4 | none | MW | 2 |
| 7 | yes | yes | no | yes | yes | no | no | yes | no | 3-4 | none | MW | 2 |
| 8 | yes | no | no | yes | no | no | yes | yes | no | 3-4 | MW | MW | 2 |
| 9 | yes | no | no | yes | no | no | no | no | no | 3-4 | MW | MW | 2 |
| 10 | yes | yes | yes | yes | yes | yes | no | yes | no | 3-4 | none | MW | 2 |
| 11 | yes | yes | yes | yes | yes | no | yes | yes | no | 3-4 | MW | MW | 2 |

^a^None = no preference; current = prefers previous treatment; MW = prefers microwave treatment over previous treatments; ^b^3% diclofenac gel; ^c^5-fluorouracil; ^d^5% imiquimod cream; ^e^topical solution of 5-fluorouracil 0.5% and salicylic acid 10%; ^f^ingenol mebutate gel

## Supplementary Table S3: Summary for primary endpoint by treatment protocol

|  |  | Participants 1 & 2 (Initial Protocol) | | | |  |  | Participants 3 – 11 (Updated Protocol) | | | |  |
| --- | --- | --- | --- | --- | --- | --- | --- | --- | --- | --- | --- | --- |
|  | Treated | | | Not Treated | | |  | Treated |  | Not Treated | | |
| Visit | No. lesions | No. responding | (%) | No. lesions | No. responding | (%) | No. lesions | No. responding | (%) | No. lesions | No. responding | (%) |
| Visit 3 (day 8) | 19 | 17 | (89%) | 19 | 1 | (5%) | 74 | 56 | (76%) | 67 | 1 | (1%) |
| Visit 4 (day 15) | 19 | 19 | (100%) | 19 | 2 | (11%) | 74 | 54 | (73%) | 67 | 5 | (7%) |
| Visit 6 (day 28) | 19 | 19 | (100%) | 19 | 2 | (11%) | 74 | 67 | (91%) | 67 | 7 | (10%) |
| Visit 8 (day 42) | 19 | 19 | (100%) | 19 | 1 | (5%) | 74 | 67 | (91%) | 67 | 8 | (12%) |
| Visit 10 (day 60) | 19 | 19 | (100%) | 19 | 3 | (16%) | 65 | 62 | (95%) | 67 | 8 | (12%) |
| Visit 11 (day 120) | 19 | 19 | (100%) | 19 | 3 | (16%) | 74 | 65 | (88%) | 67 | 10 | (15%) |
