# Supplementary material for: A feasibility study of microwave therapy for precancerous actinic keratosis
Source: Br J Dermatol. 2020 Mar 23;183(2):222–30. doi: 10.1111/bjd.18935 (PMC7496712; doi:10.1111/bjd.18935)
Supplement: Supplementary file 2 — Powerpoint S1 Journal Club Slide Set. [file BJD-183-222-s002.pptx]

## Slide 1
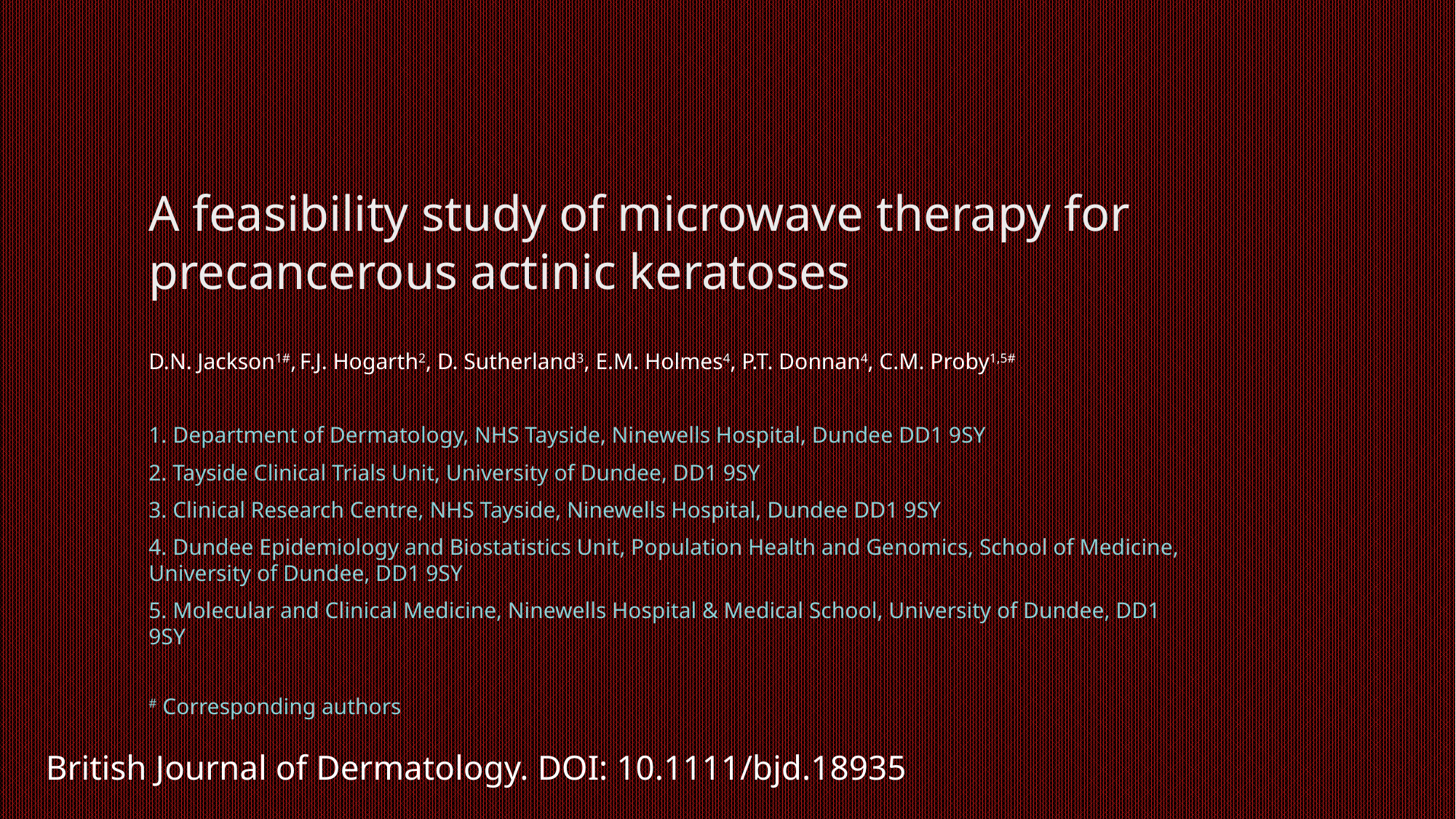

# A feasibility study of microwave therapy for precancerous actinic keratoses
D.N. Jackson1#, F.J. Hogarth2, D. Sutherland3, E.M. Holmes4, P.T. Donnan4, C.M. Proby1,5#
1. Department of Dermatology, NHS Tayside, Ninewells Hospital, Dundee DD1 9SY
2. Tayside Clinical Trials Unit, University of Dundee, DD1 9SY
3. Clinical Research Centre, NHS Tayside, Ninewells Hospital, Dundee DD1 9SY
4. Dundee Epidemiology and Biostatistics Unit, Population Health and Genomics, School of Medicine, University of Dundee, DD1 9SY
5. Molecular and Clinical Medicine, Ninewells Hospital & Medical School, University of Dundee, DD1 9SY
# Corresponding authors
British Journal of Dermatology. DOI: 10.1111/bjd.18935

## Slide 2
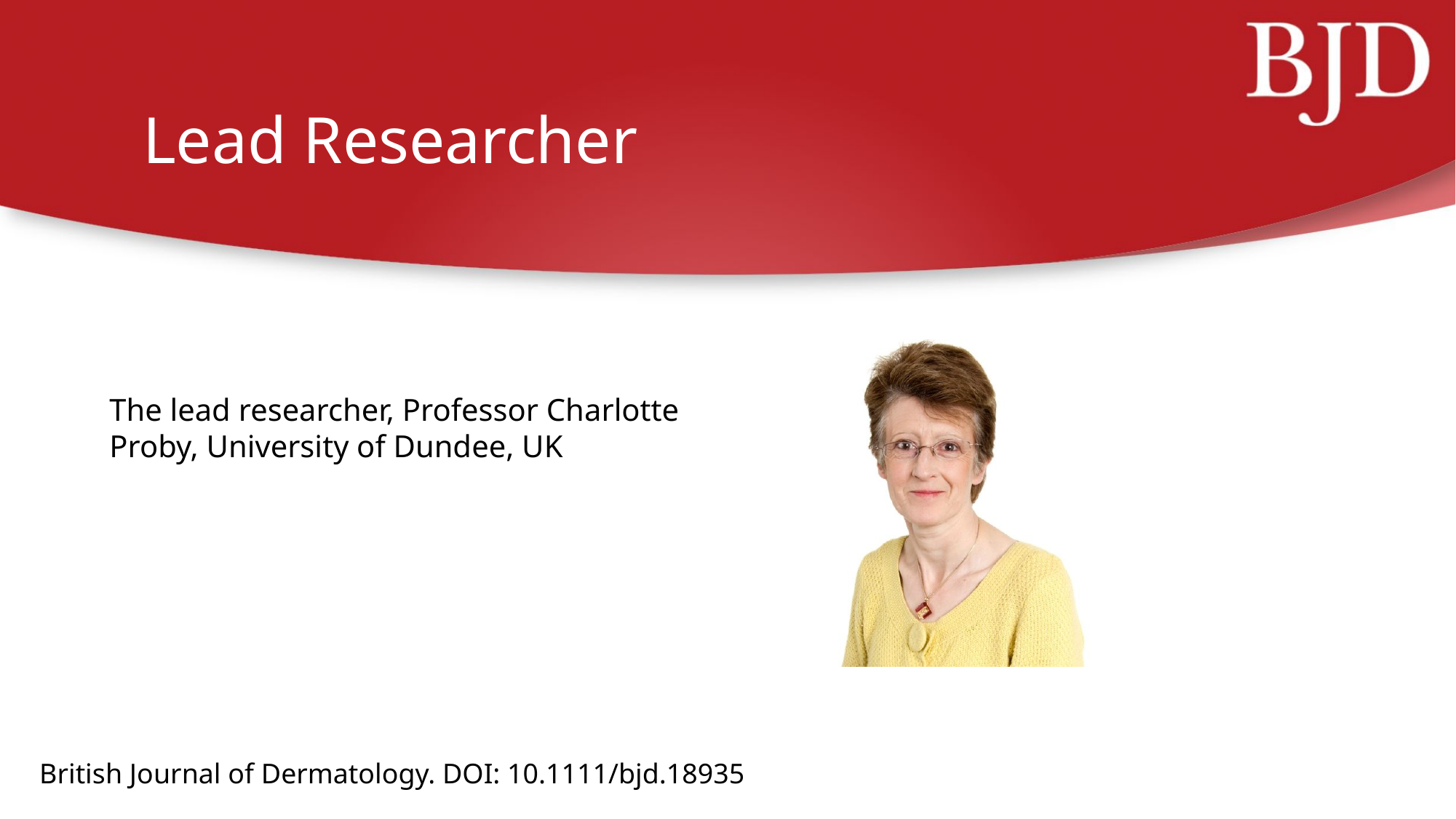

# Lead Researcher
The lead researcher, Professor Charlotte Proby, University of Dundee, UK
British Journal of Dermatology. DOI: 10.1111/bjd.18935

## Slide 3
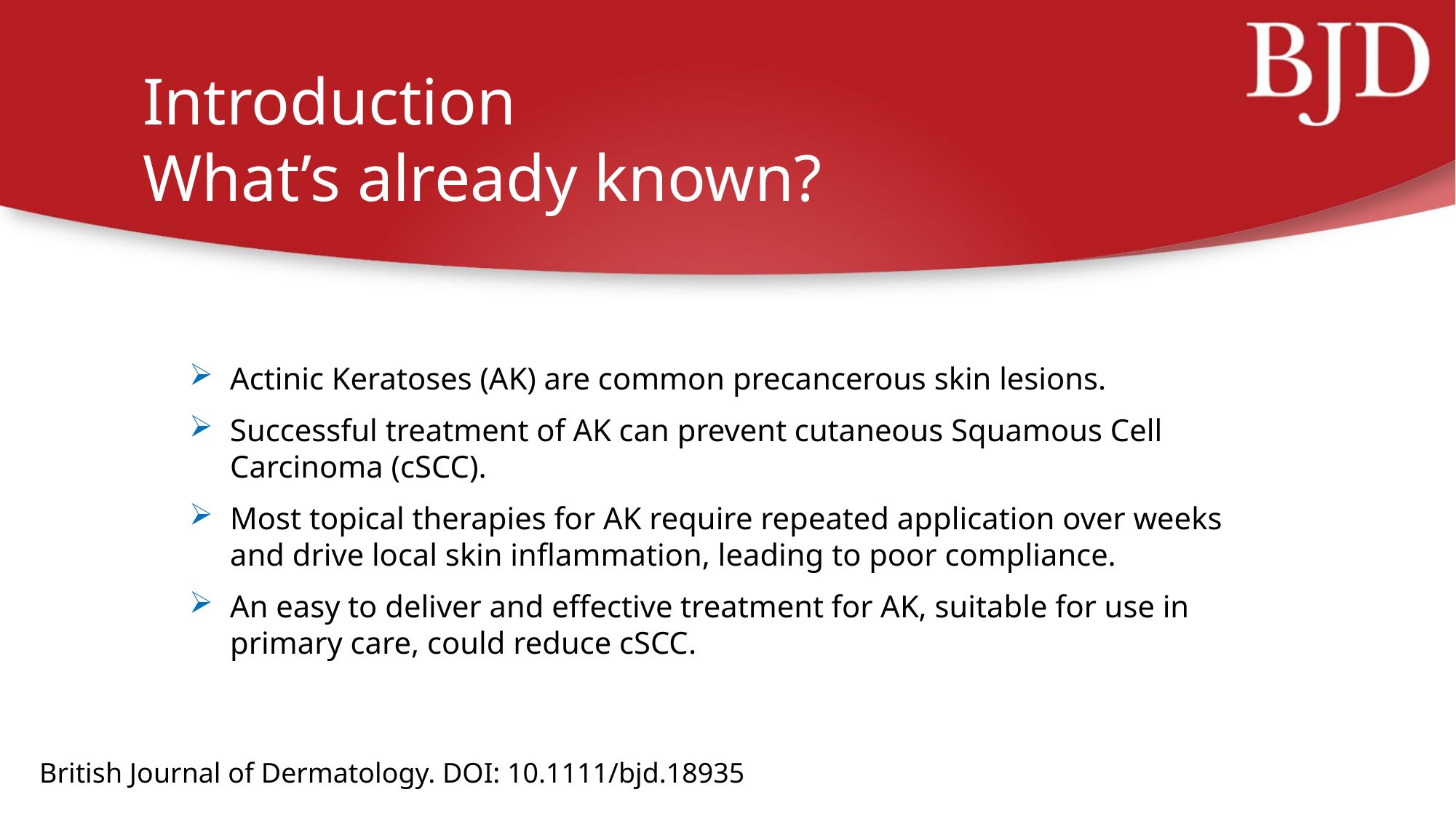

# Introduction What’s already known?
Actinic Keratoses (AK) are common precancerous skin lesions.
Successful treatment of AK can prevent cutaneous Squamous Cell Carcinoma (cSCC).
Most topical therapies for AK require repeated application over weeks and drive local skin inflammation, leading to poor compliance.
An easy to deliver and effective treatment for AK, suitable for use in primary care, could reduce cSCC.
British Journal of Dermatology. DOI: 10.1111/bjd.18935

## Slide 4
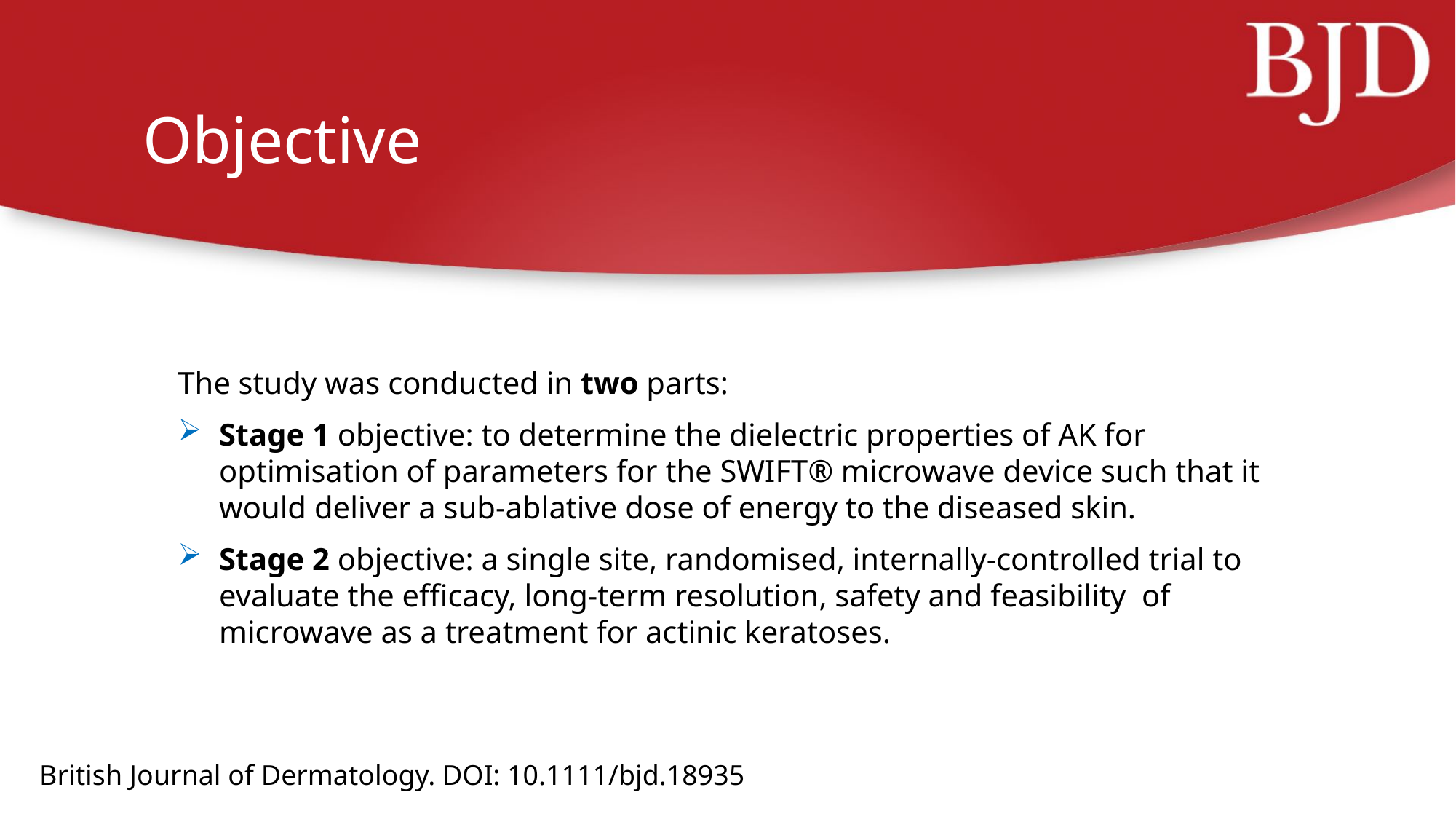

# Objective
The study was conducted in two parts:
Stage 1 objective: to determine the dielectric properties of AK for optimisation of parameters for the SWIFT® microwave device such that it would deliver a sub-ablative dose of energy to the diseased skin.
Stage 2 objective: a single site, randomised, internally-controlled trial to evaluate the efficacy, long-term resolution, safety and feasibility of microwave as a treatment for actinic keratoses.
British Journal of Dermatology. DOI: 10.1111/bjd.18935

## Slide 5
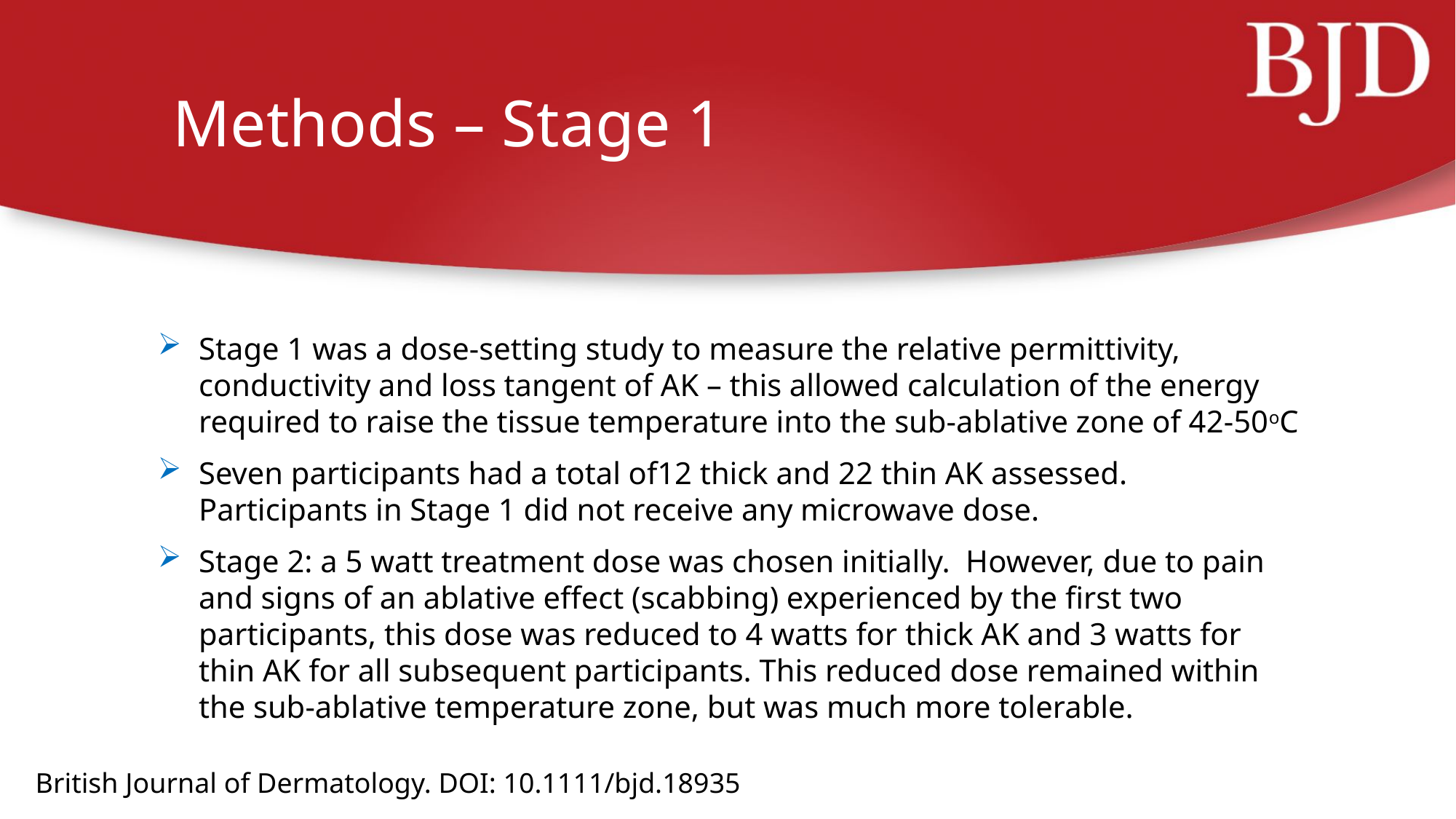

# Methods – Stage 1
Stage 1 was a dose-setting study to measure the relative permittivity, conductivity and loss tangent of AK – this allowed calculation of the energy required to raise the tissue temperature into the sub-ablative zone of 42-50oC
Seven participants had a total of12 thick and 22 thin AK assessed. Participants in Stage 1 did not receive any microwave dose.
Stage 2: a 5 watt treatment dose was chosen initially. However, due to pain and signs of an ablative effect (scabbing) experienced by the first two participants, this dose was reduced to 4 watts for thick AK and 3 watts for thin AK for all subsequent participants. This reduced dose remained within the sub-ablative temperature zone, but was much more tolerable.
British Journal of Dermatology. DOI: 10.1111/bjd.18935

## Slide 6
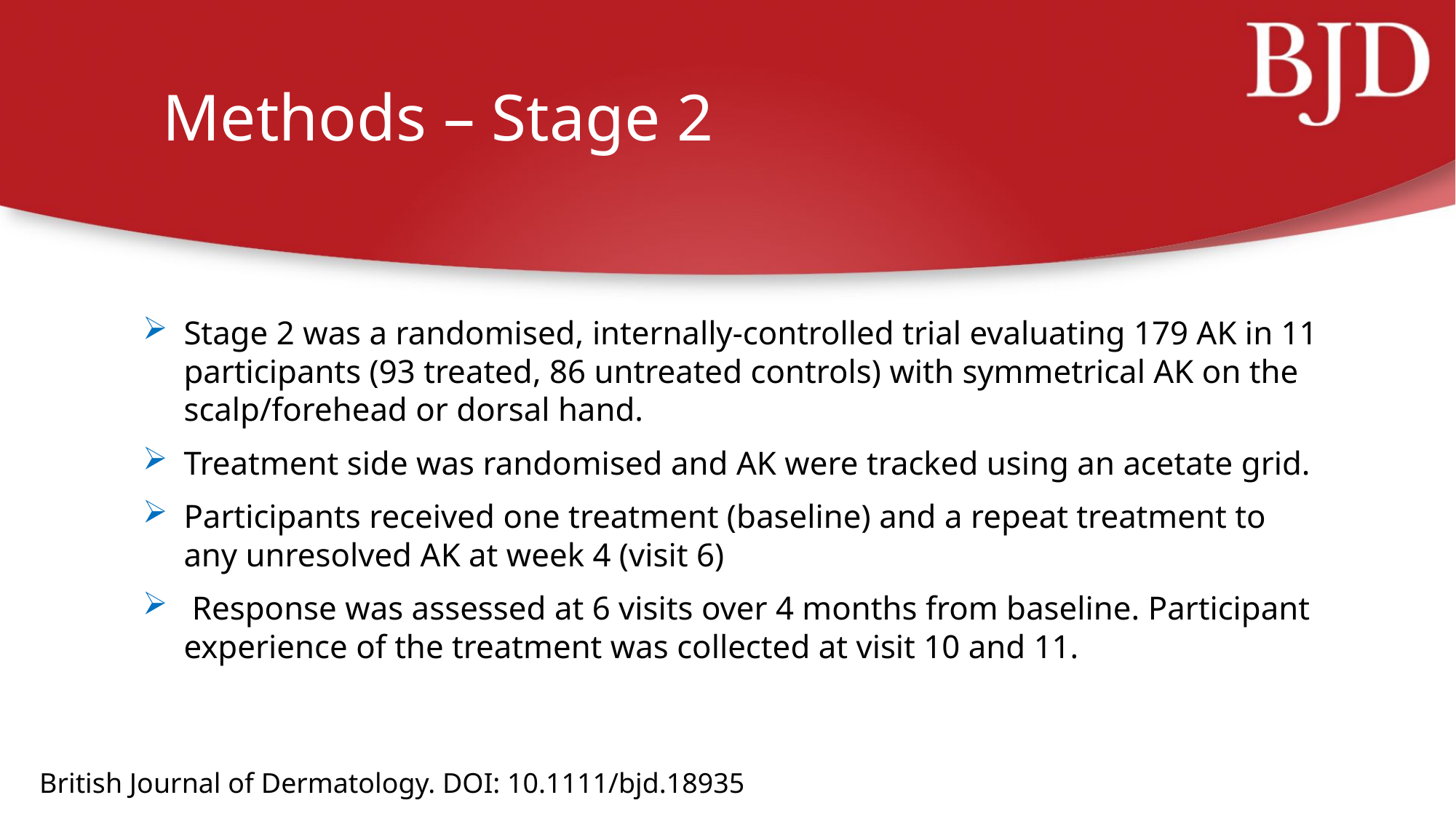

# Methods – Stage 2
Stage 2 was a randomised, internally-controlled trial evaluating 179 AK in 11 participants (93 treated, 86 untreated controls) with symmetrical AK on the scalp/forehead or dorsal hand.
Treatment side was randomised and AK were tracked using an acetate grid.
Participants received one treatment (baseline) and a repeat treatment to any unresolved AK at week 4 (visit 6)
 Response was assessed at 6 visits over 4 months from baseline. Participant experience of the treatment was collected at visit 10 and 11.
British Journal of Dermatology. DOI: 10.1111/bjd.18935

## Slide 7
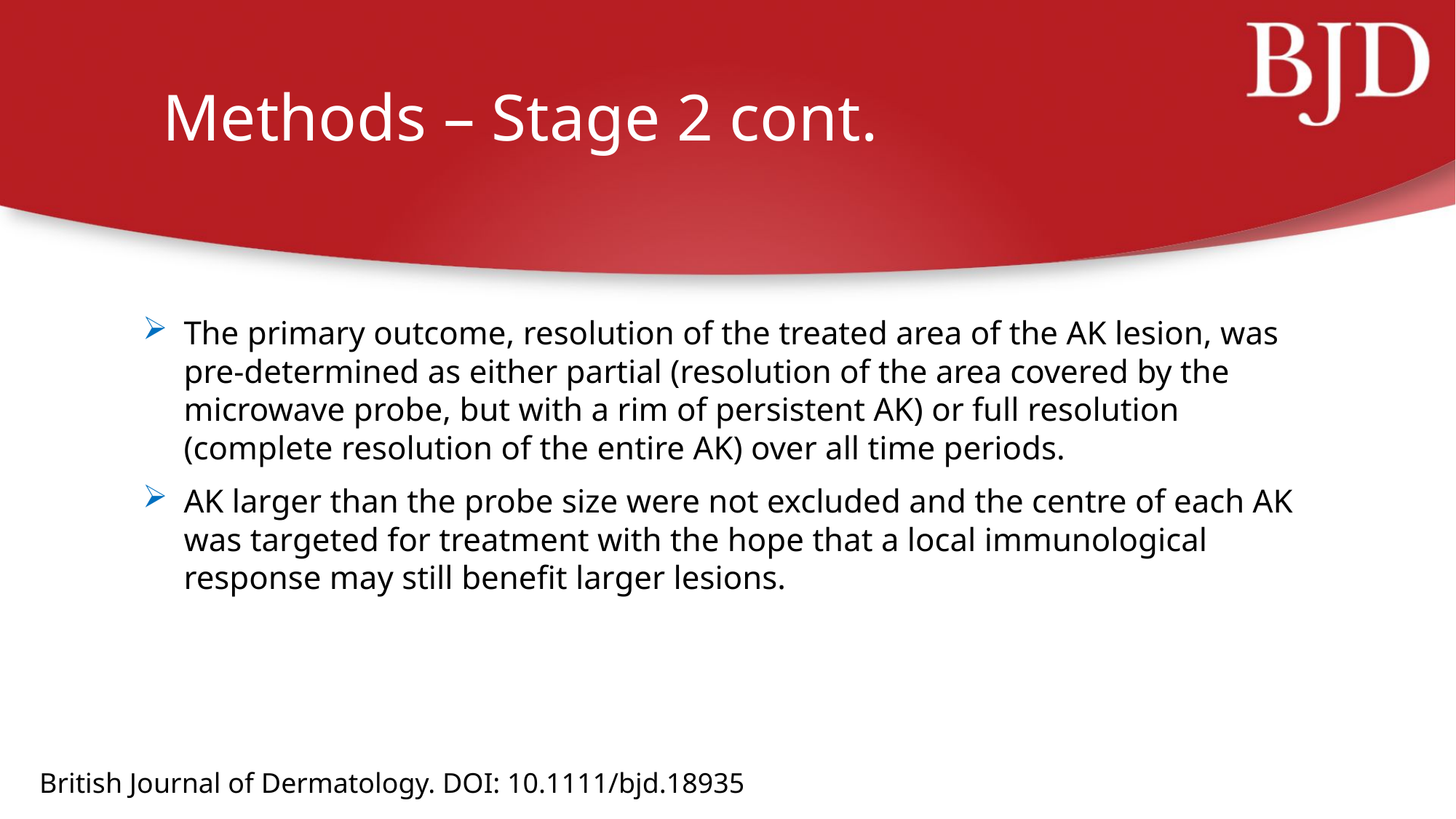

# Methods – Stage 2 cont.
The primary outcome, resolution of the treated area of the AK lesion, was pre-determined as either partial (resolution of the area covered by the microwave probe, but with a rim of persistent AK) or full resolution (complete resolution of the entire AK) over all time periods.
AK larger than the probe size were not excluded and the centre of each AK was targeted for treatment with the hope that a local immunological response may still benefit larger lesions.
British Journal of Dermatology. DOI: 10.1111/bjd.18935

## Slide 8
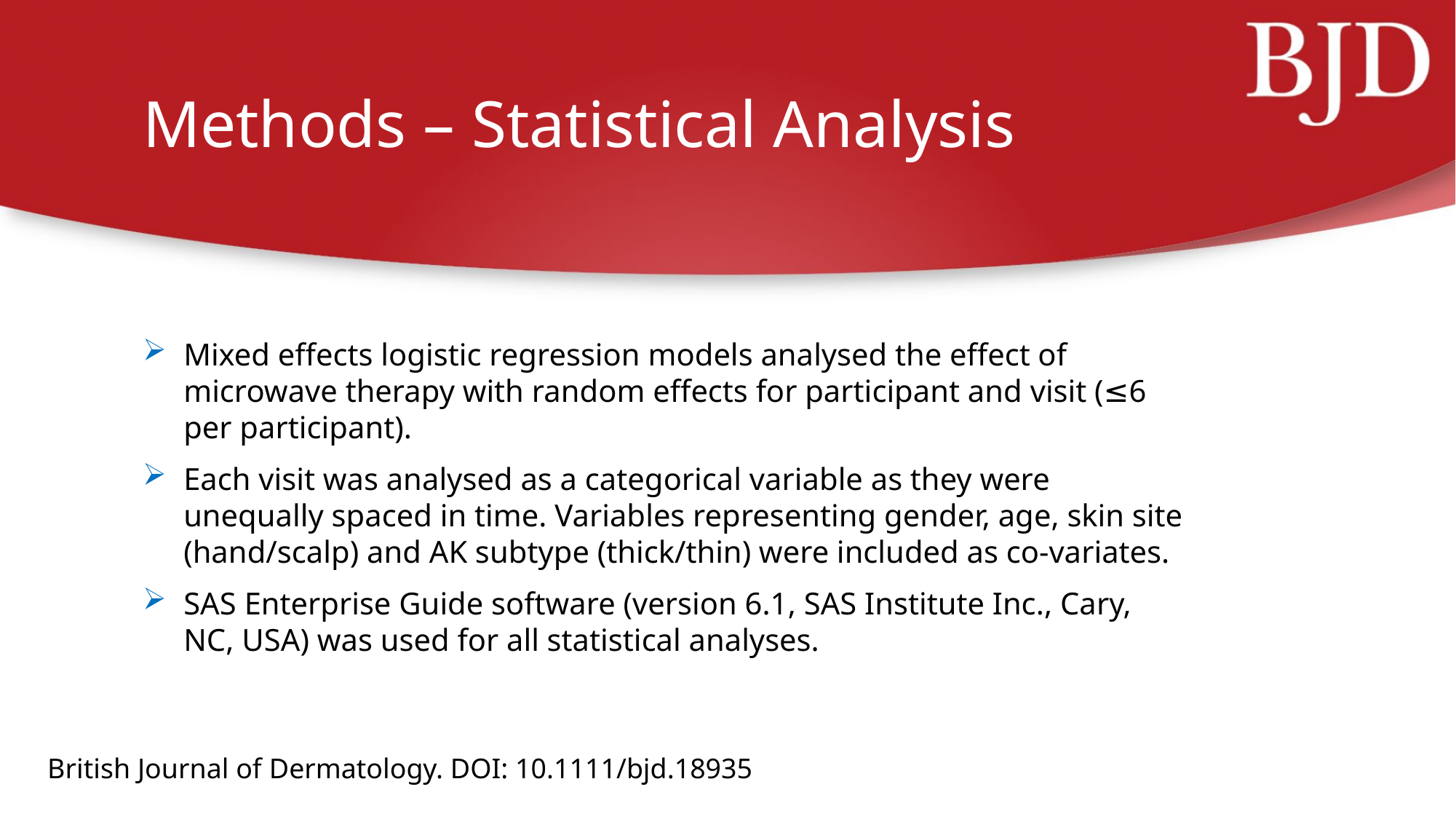

# Methods – Statistical Analysis
Mixed effects logistic regression models analysed the effect of microwave therapy with random effects for participant and visit (≤6 per participant).
Each visit was analysed as a categorical variable as they were unequally spaced in time. Variables representing gender, age, skin site (hand/scalp) and AK subtype (thick/thin) were included as co-variates.
SAS Enterprise Guide software (version 6.1, SAS Institute Inc., Cary, NC, USA) was used for all statistical analyses.
British Journal of Dermatology. DOI: 10.1111/bjd.18935

## Slide 9
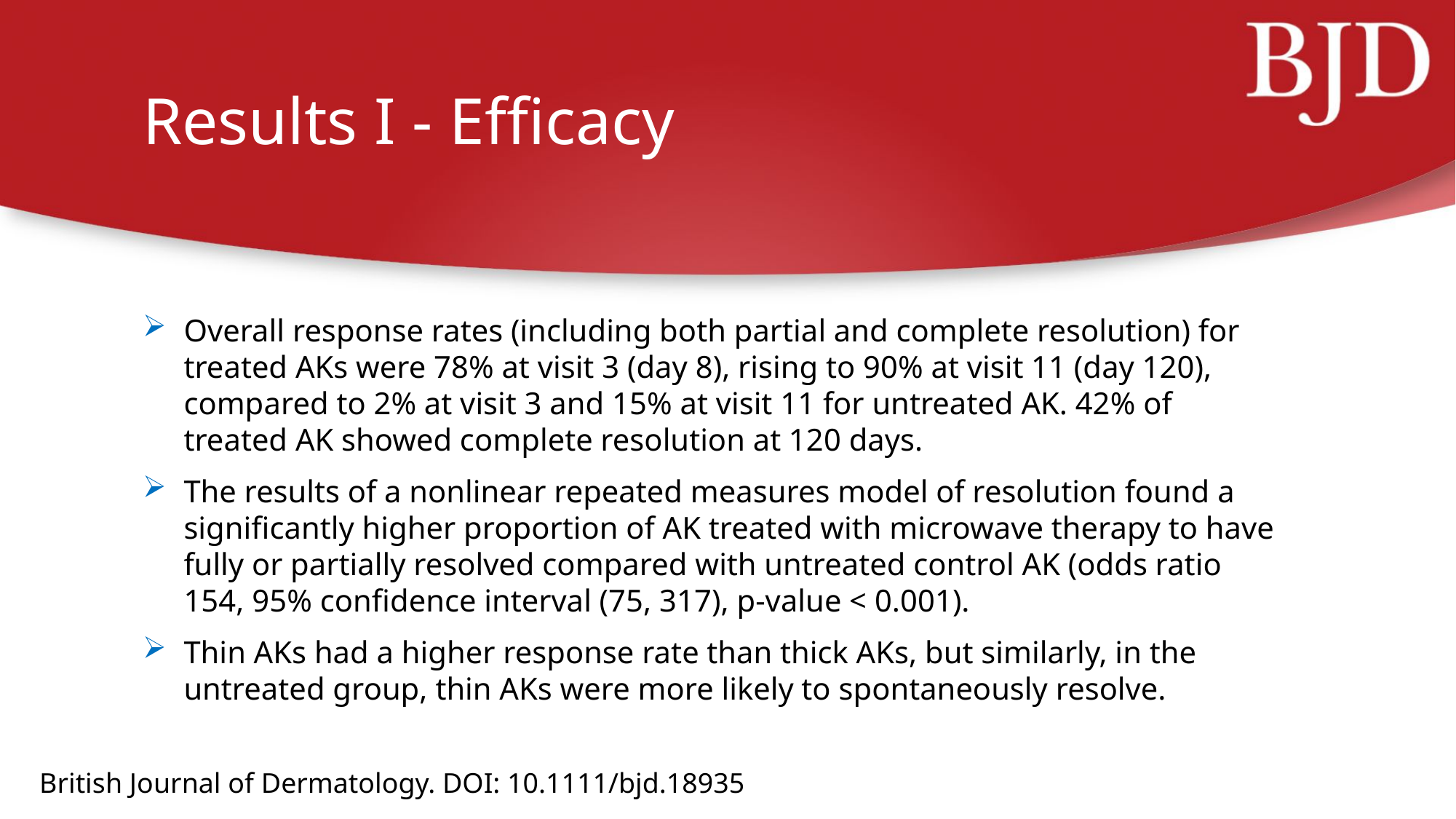

# Results I - Efficacy
Overall response rates (including both partial and complete resolution) for treated AKs were 78% at visit 3 (day 8), rising to 90% at visit 11 (day 120), compared to 2% at visit 3 and 15% at visit 11 for untreated AK. 42% of treated AK showed complete resolution at 120 days.
The results of a nonlinear repeated measures model of resolution found a significantly higher proportion of AK treated with microwave therapy to have fully or partially resolved compared with untreated control AK (odds ratio 154, 95% confidence interval (75, 317), p-value < 0.001).
Thin AKs had a higher response rate than thick AKs, but similarly, in the untreated group, thin AKs were more likely to spontaneously resolve.
British Journal of Dermatology. DOI: 10.1111/bjd.18935

## Slide 10
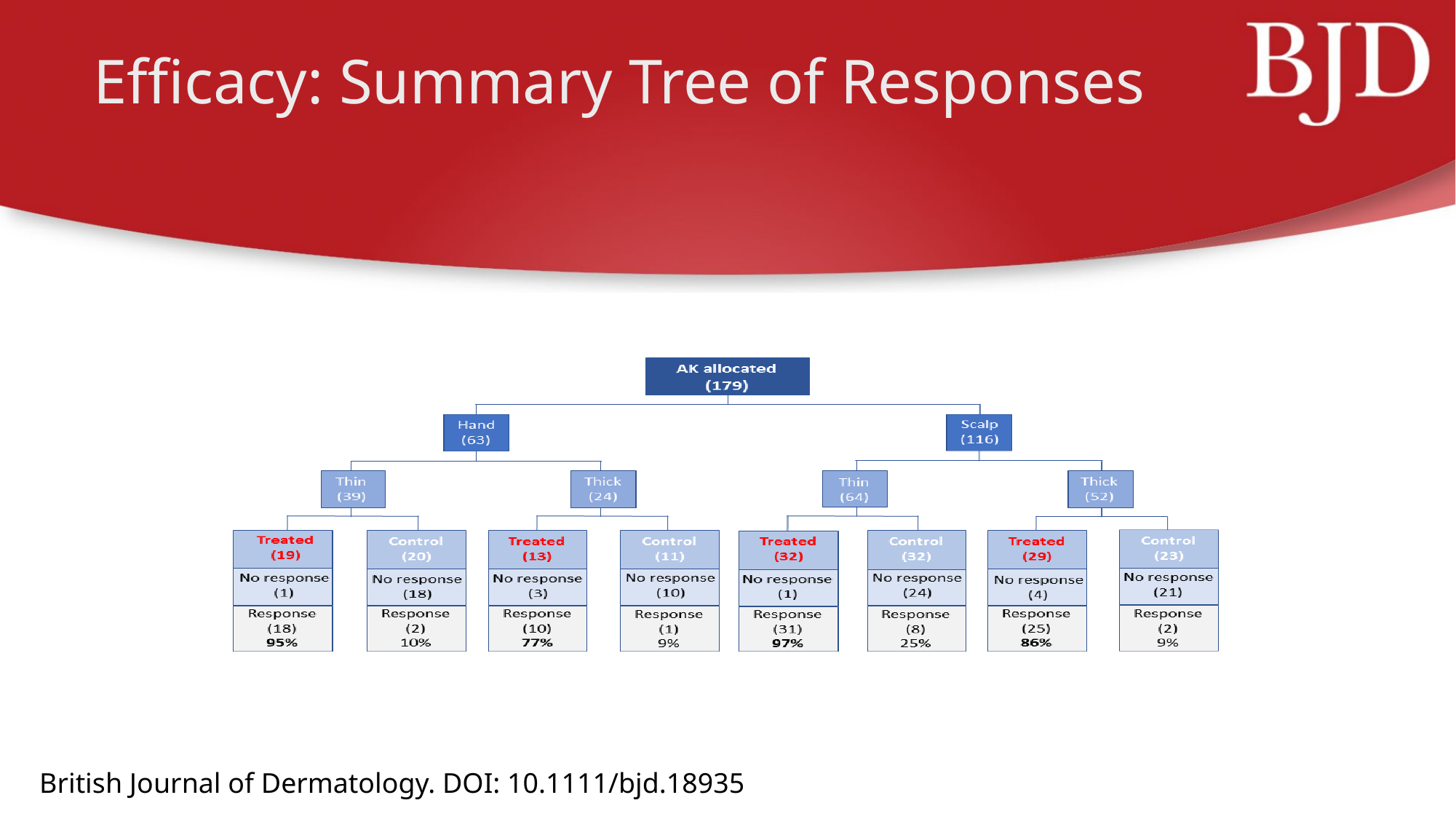

# Efficacy: Summary Tree of Responses
British Journal of Dermatology. DOI: 10.1111/bjd.18935

## Slide 11
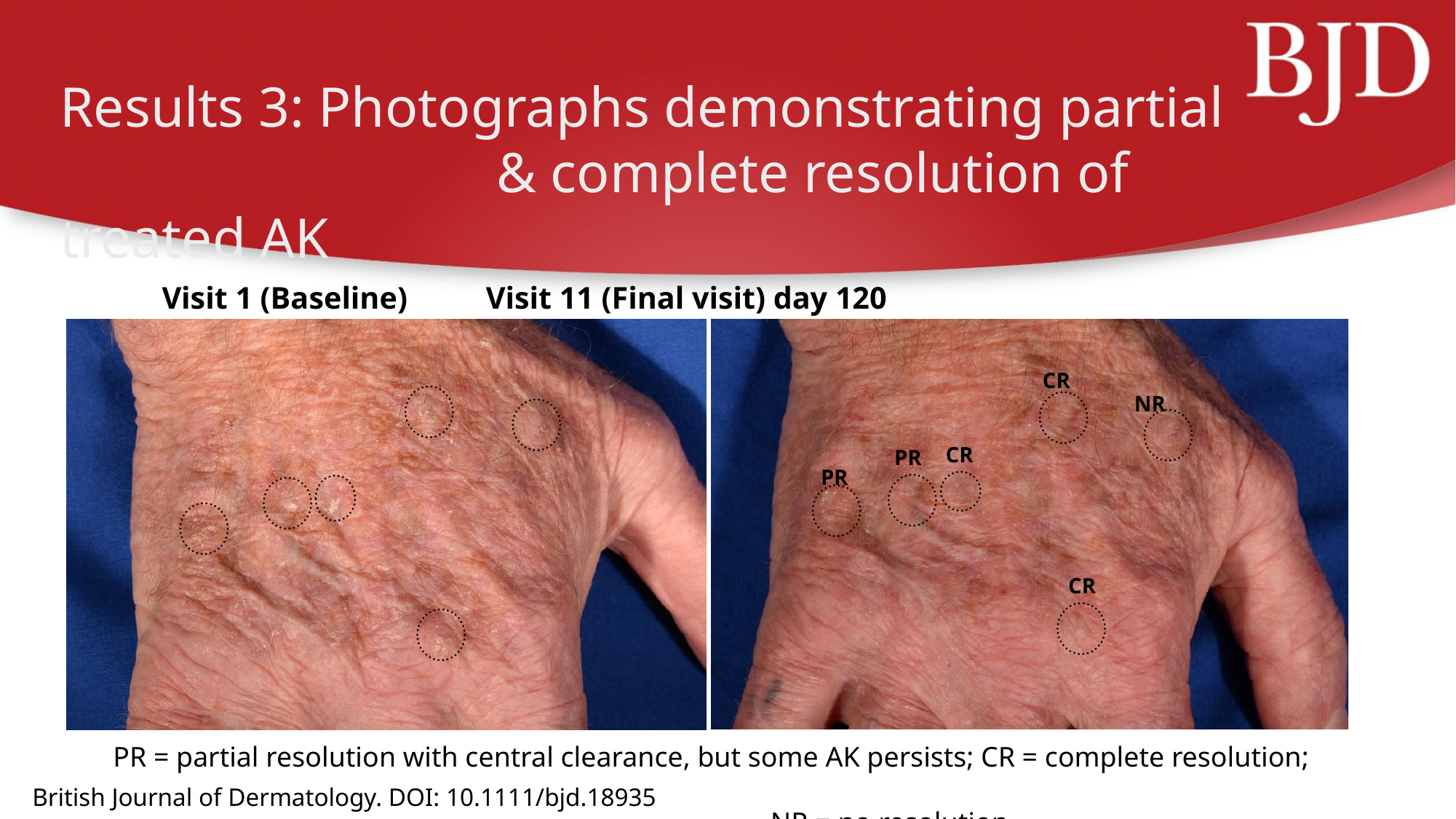

# Results 3: Photographs demonstrating partial 				& complete resolution of treated AK
Visit 1 (Baseline)										Visit 11 (Final visit) day 120
CR
NR
CR
PR
PR
CR
PR = partial resolution with central clearance, but some AK persists; CR = complete resolution;
														 NR = no resolution
British Journal of Dermatology. DOI: 10.1111/bjd.18935

## Slide 12
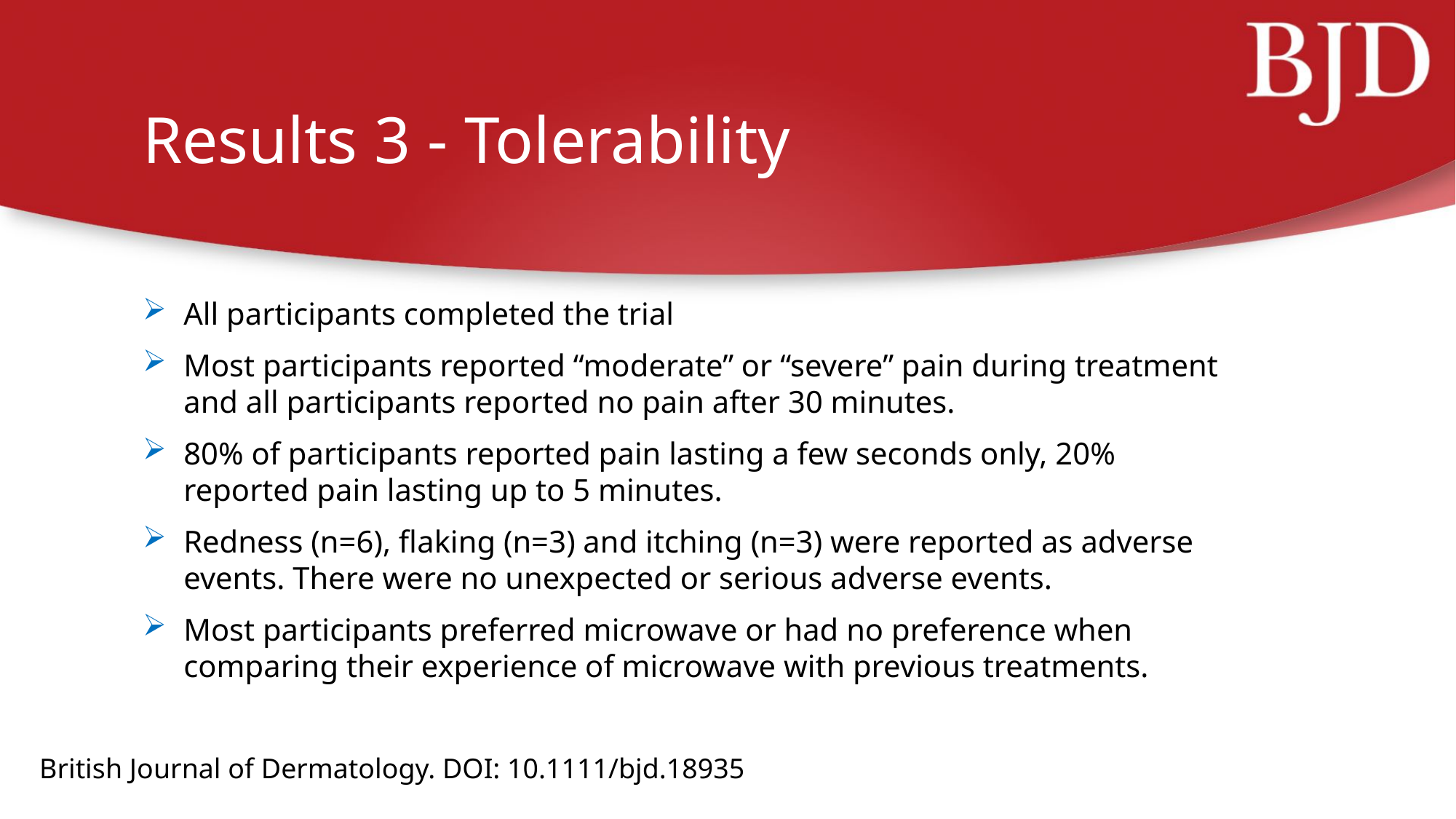

# Results 3 - Tolerability
All participants completed the trial
Most participants reported “moderate” or “severe” pain during treatment and all participants reported no pain after 30 minutes.
80% of participants reported pain lasting a few seconds only, 20% reported pain lasting up to 5 minutes.
Redness (n=6), flaking (n=3) and itching (n=3) were reported as adverse events. There were no unexpected or serious adverse events.
Most participants preferred microwave or had no preference when comparing their experience of microwave with previous treatments.
British Journal of Dermatology. DOI: 10.1111/bjd.18935

## Slide 13
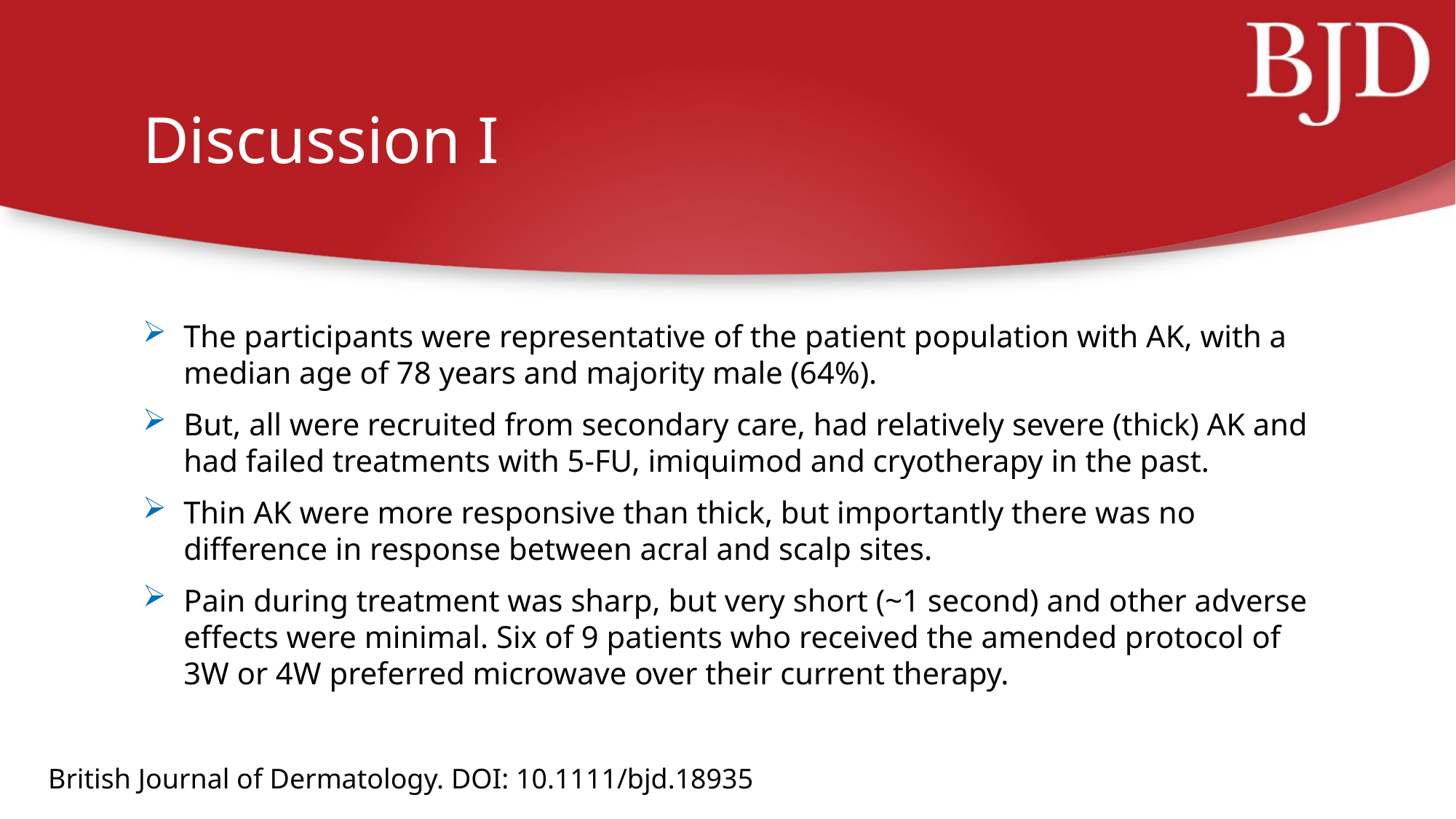

# Discussion I
The participants were representative of the patient population with AK, with a median age of 78 years and majority male (64%).
But, all were recruited from secondary care, had relatively severe (thick) AK and had failed treatments with 5-FU, imiquimod and cryotherapy in the past.
Thin AK were more responsive than thick, but importantly there was no difference in response between acral and scalp sites.
Pain during treatment was sharp, but very short (~1 second) and other adverse effects were minimal. Six of 9 patients who received the amended protocol of 3W or 4W preferred microwave over their current therapy.
British Journal of Dermatology. DOI: 10.1111/bjd.18935

## Slide 14
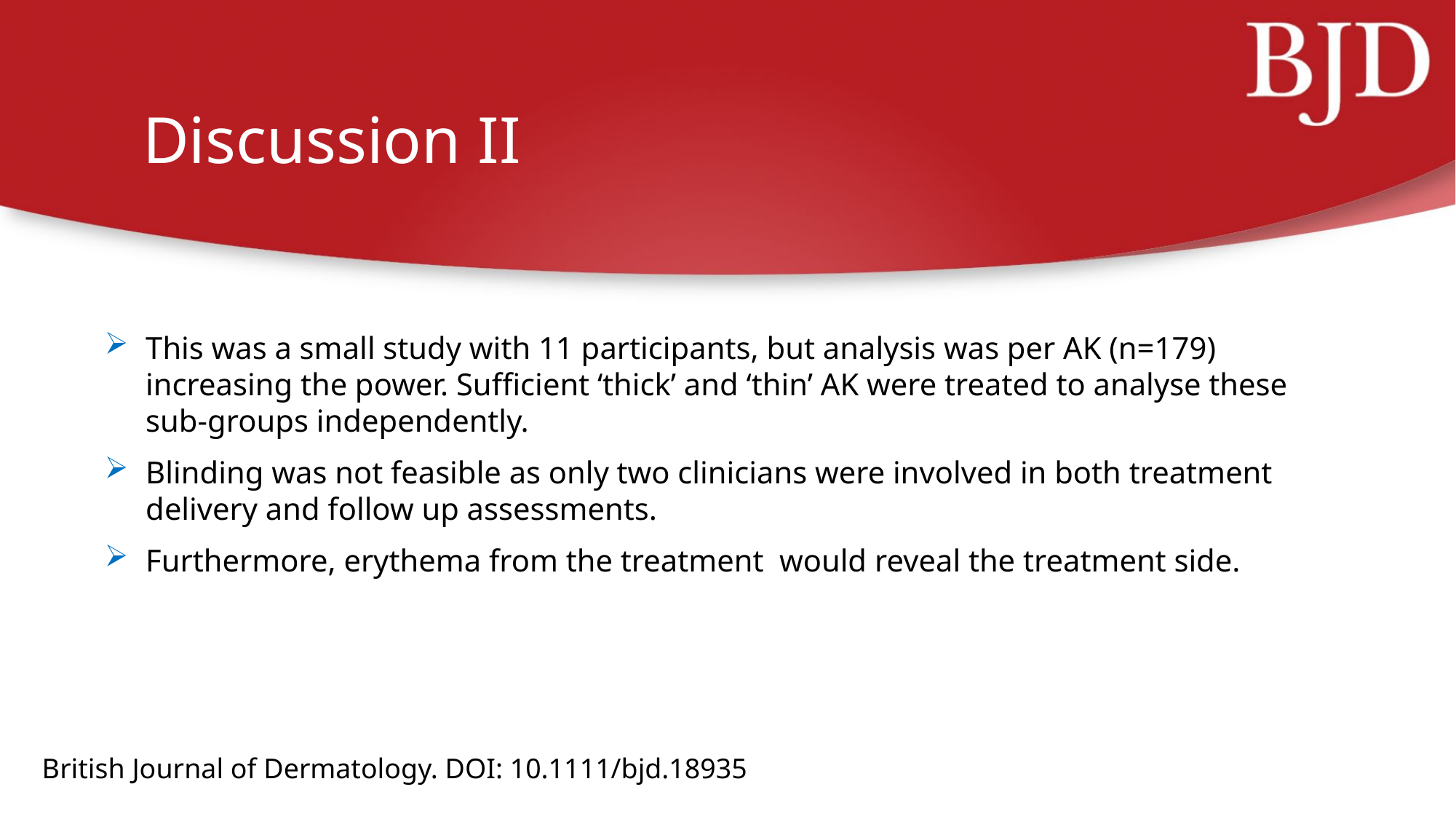

# Discussion II
This was a small study with 11 participants, but analysis was per AK (n=179) increasing the power. Sufficient ‘thick’ and ‘thin’ AK were treated to analyse these sub-groups independently.
Blinding was not feasible as only two clinicians were involved in both treatment delivery and follow up assessments.
Furthermore, erythema from the treatment would reveal the treatment side.
British Journal of Dermatology. DOI: 10.1111/bjd.18935

## Slide 15
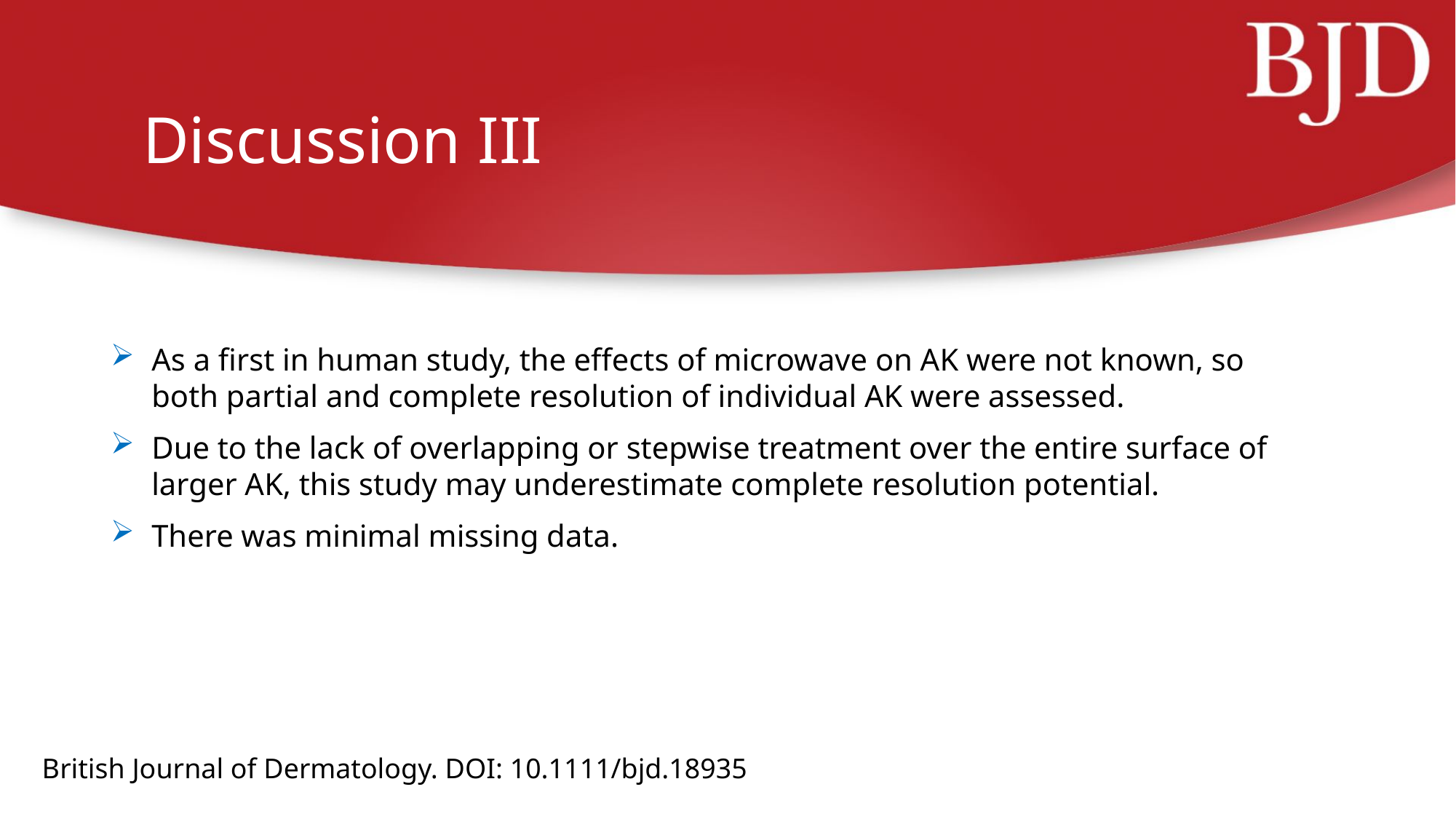

# Discussion III
As a first in human study, the effects of microwave on AK were not known, so both partial and complete resolution of individual AK were assessed.
Due to the lack of overlapping or stepwise treatment over the entire surface of larger AK, this study may underestimate complete resolution potential.
There was minimal missing data.
British Journal of Dermatology. DOI: 10.1111/bjd.18935

## Slide 16
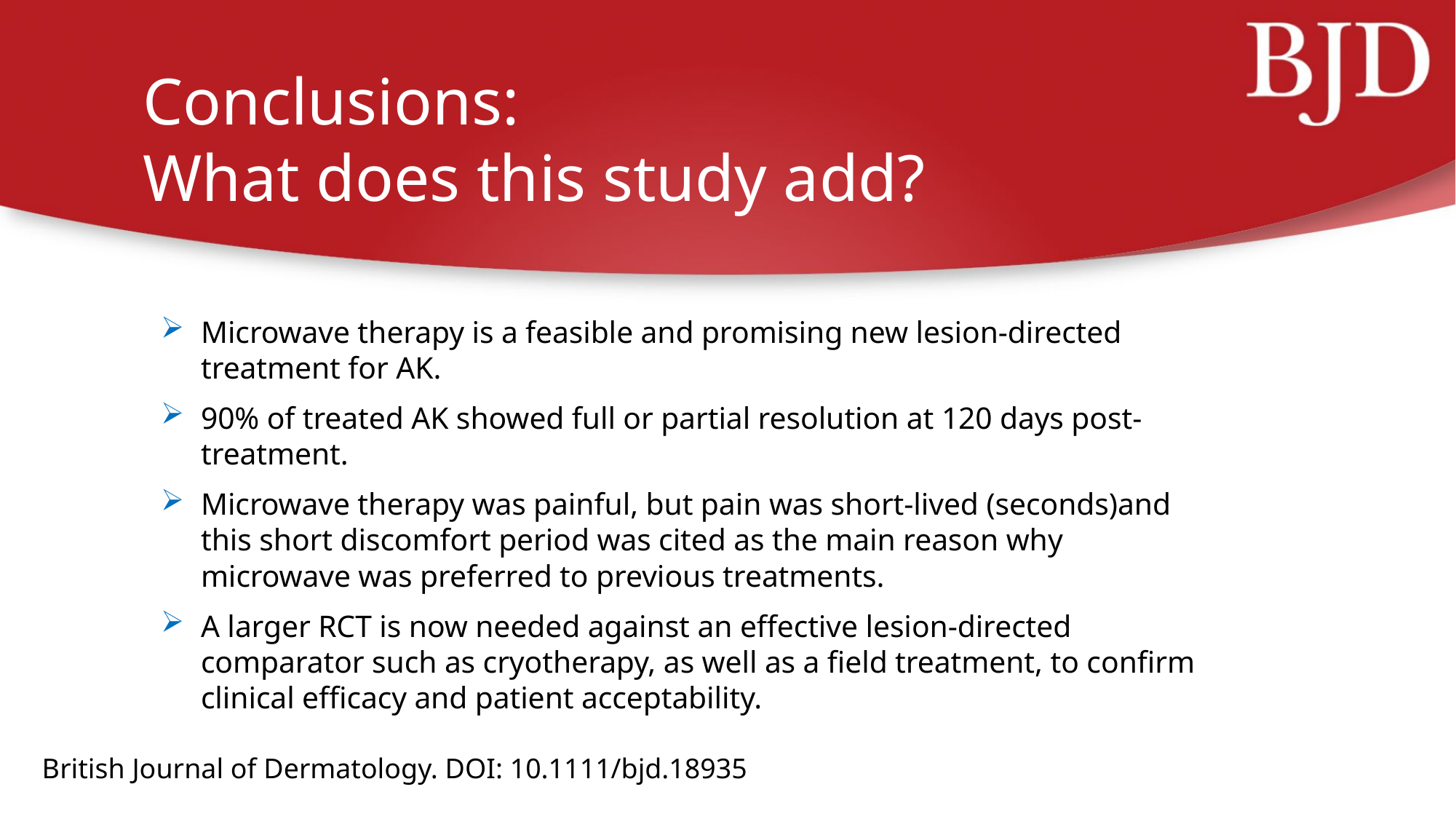

# Conclusions: What does this study add?
Microwave therapy is a feasible and promising new lesion-directed treatment for AK.
90% of treated AK showed full or partial resolution at 120 days post-treatment.
Microwave therapy was painful, but pain was short-lived (seconds)and this short discomfort period was cited as the main reason why microwave was preferred to previous treatments.
A larger RCT is now needed against an effective lesion-directed comparator such as cryotherapy, as well as a field treatment, to confirm clinical efficacy and patient acceptability.
British Journal of Dermatology. DOI: 10.1111/bjd.18935

## Slide 17
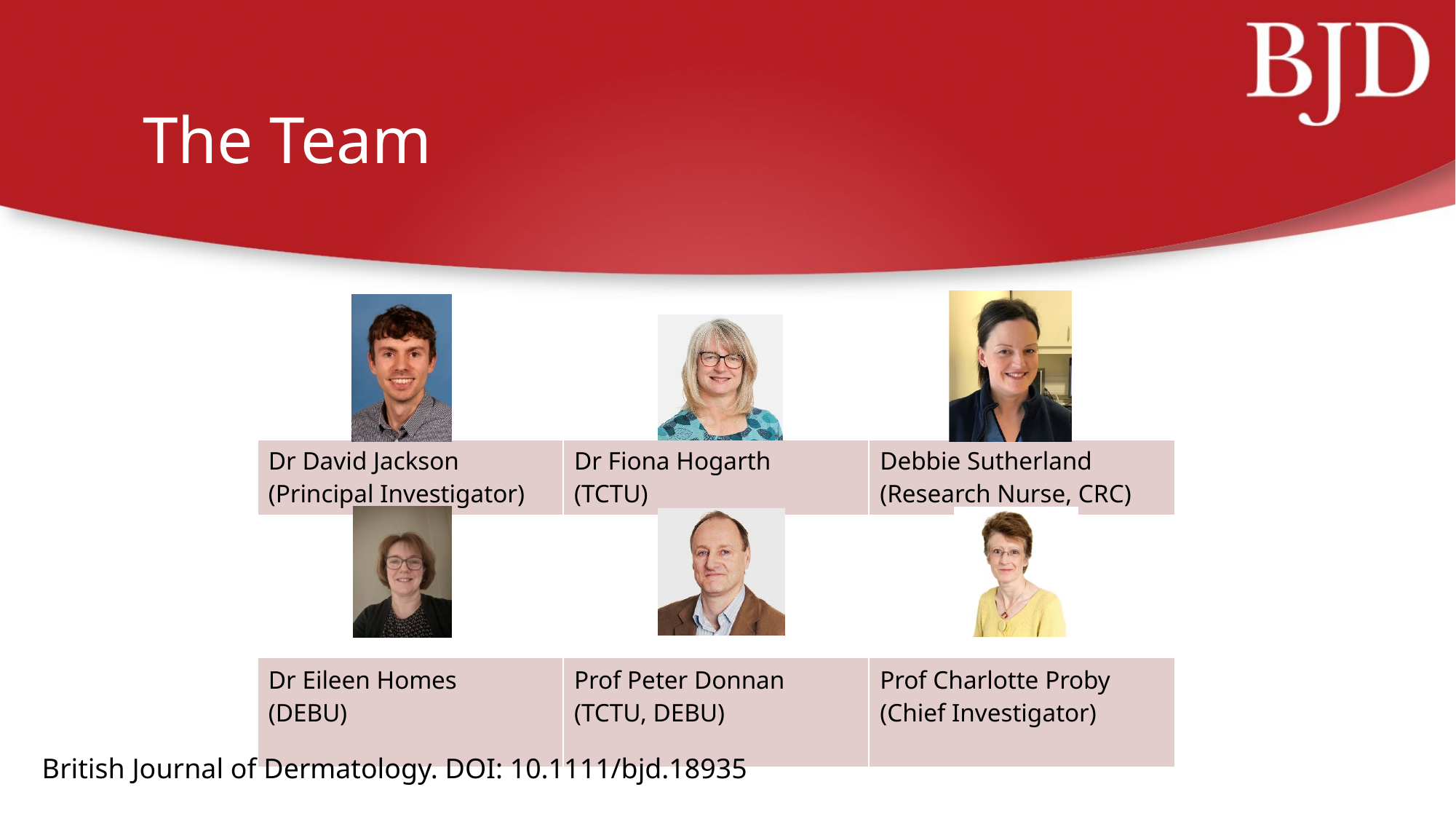

# The Team
| | | |
| --- | --- | --- |
| Dr David Jackson (Principal Investigator) | Dr Fiona Hogarth (TCTU) | Debbie Sutherland (Research Nurse, CRC) |
| | | |
| Dr Eileen Homes (DEBU) | Prof Peter Donnan (TCTU, DEBU) | Prof Charlotte Proby (Chief Investigator) |
British Journal of Dermatology. DOI: 10.1111/bjd.18935

## Slide 18
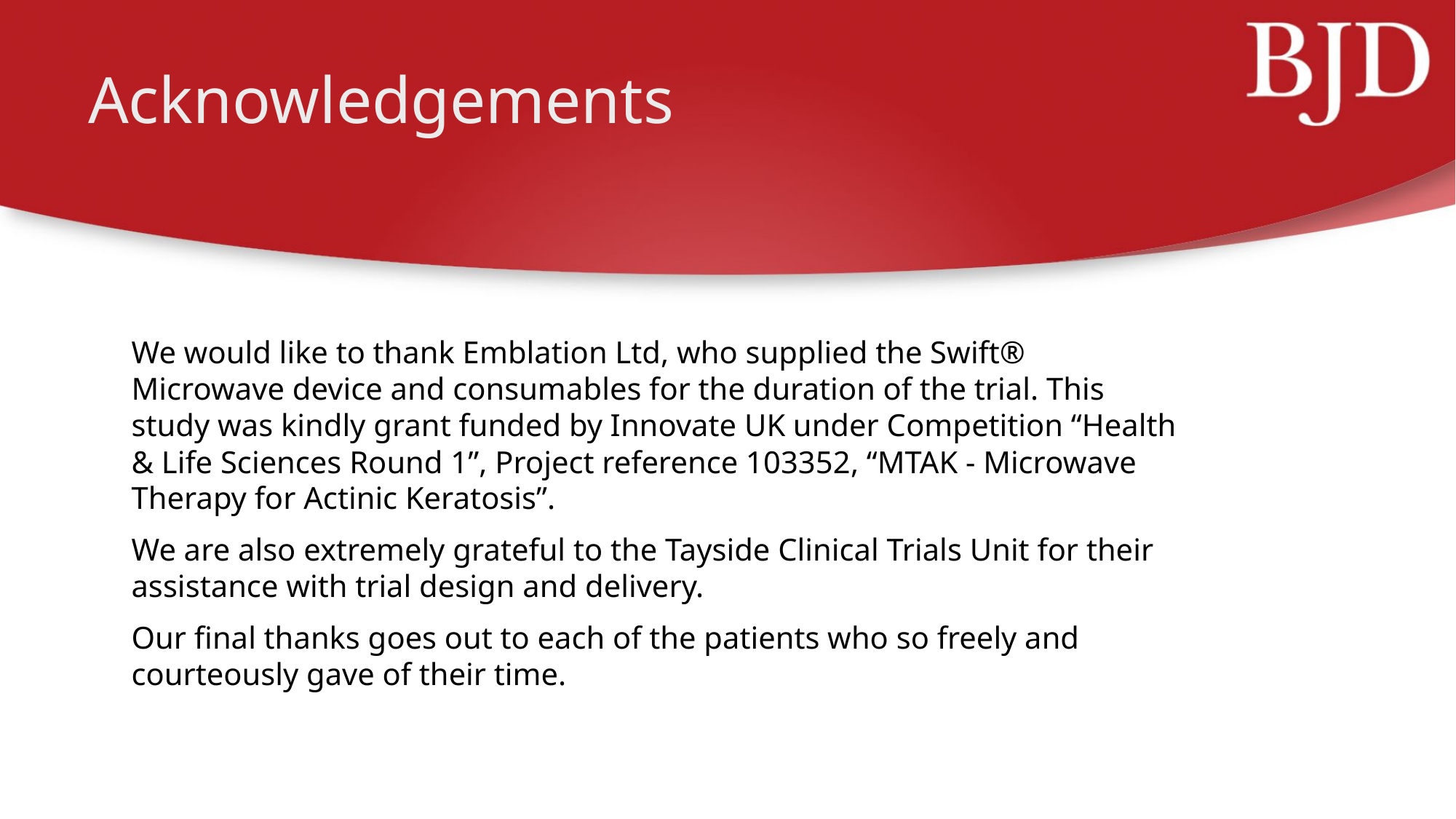

# Acknowledgements
We would like to thank Emblation Ltd, who supplied the Swift® Microwave device and consumables for the duration of the trial. This study was kindly grant funded by Innovate UK under Competition “Health & Life Sciences Round 1”, Project reference 103352, “MTAK - Microwave Therapy for Actinic Keratosis”.
We are also extremely grateful to the Tayside Clinical Trials Unit for their assistance with trial design and delivery.
Our final thanks goes out to each of the patients who so freely and courteously gave of their time.

## Slide 19
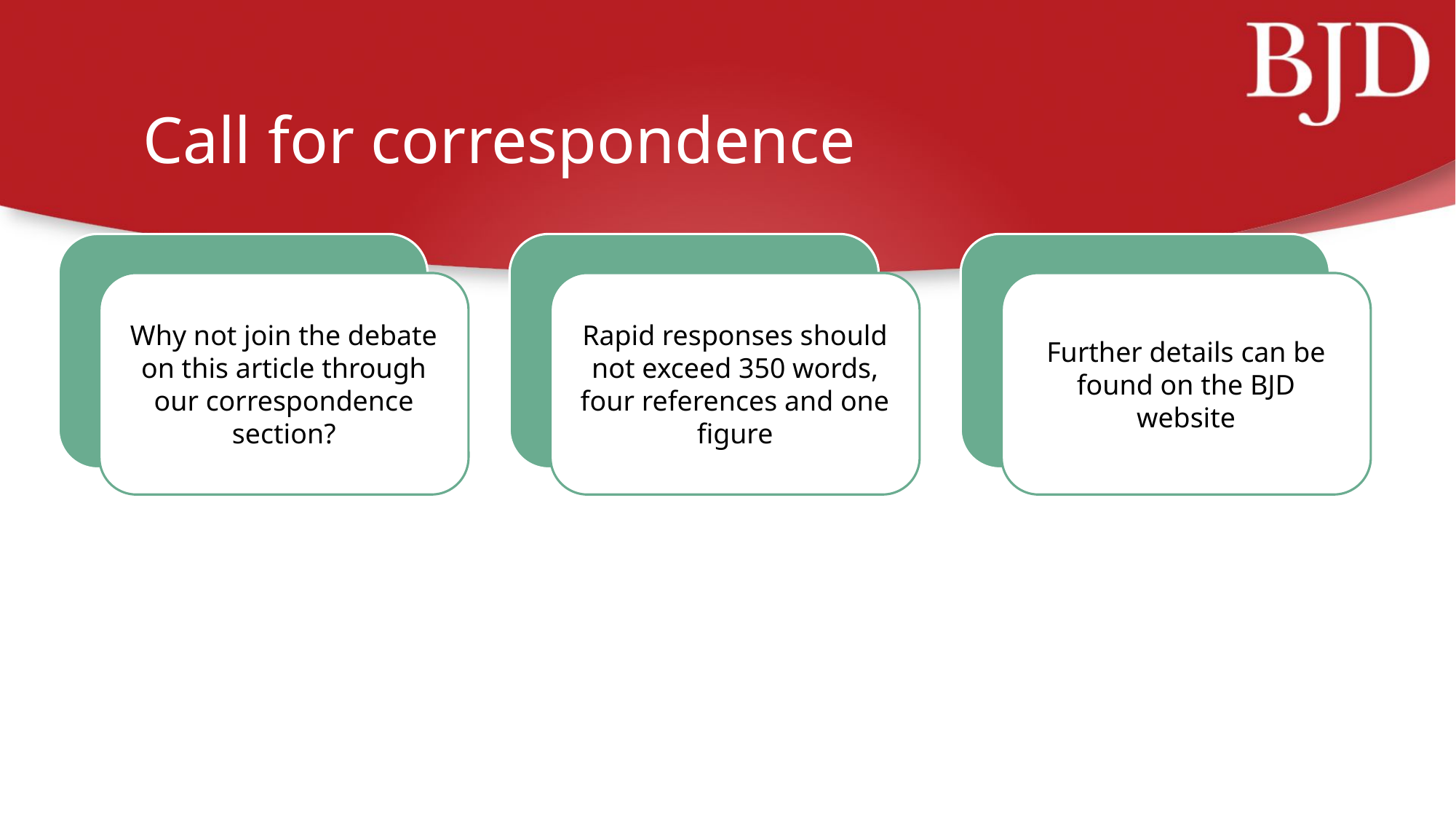

# Call for correspondence
